# Development of an AI-Driven Computational Framework for Integrated Dietary Pattern Assessment: A Simulation-Based Proof-of-Concept Study

**DOI:** 10.3390/nu18030535

**Published:** 2026-02-05

**Authors:** Mohammad Fazle Rabbi

**Affiliations:** Coordination and Research Centre for Social Sciences, Faculty of Economics and Business, University of Debrecen, Böszörményi út 138, 4032 Debrecen, Hungary; drrabbikhan@gmail.com or rabbi.mohammad@econ.unideb.hu

**Keywords:** artificial intelligence, dietary patterns, nutrient adequacy, environmental footprint, machine learning

## Abstract

**Background/Objectives**: Contemporary food systems face dual imperatives of ensuring nutritional adequacy while minimizing environmental resource consumption, yet conventional dietary assessment methodologies inadequately integrate these competing objectives. This simulation-based proof-of-concept study developed an artificial intelligence-driven computational framework synthesizing nutritional evaluation, environmental footprint quantification, and economic accessibility assessment. **Methods**: The analytical architecture integrated random forest classification, dimensionality reduction, and scenario-based optimization across a simulated population cohort of 1500 individuals. Food composition data encompassed 55 representative foods across eight categories linked with greenhouse gas emissions, water use, and price parameters. Four dietary patterns (Mediterranean, Western, Plant-based, Mixed) were characterized across nutrient adequacy, greenhouse gas emissions, water consumption, and economic cost. **Results**: Random forest classification achieved 39.1% accuracy, with cost, greenhouse gas emissions, and water consumption emerging as the most discriminating features. Dietary patterns exhibited convergent macronutrient profiles (protein 108.8–112.8 g per day, 4% variation) despite categorical distinctions, while calcium inadequacy pervaded all patterns (867–927.5 mg per day, 7–13% below requirements). Environmental footprints demonstrated limited differentiation (greenhouse gas 3.73–3.96 kg CO_2_e per day, 6% range). Bootstrap resampling (*n* = 1000) confirmed narrow confidence intervals, with NHANES validation revealing substantial energy intake deviations (38–58% above observed means) attributable to adequacy-prioritized design rather than observed consumption patterns. Scenario modeling identified seasonally flexible dietary configurations maintaining micronutrient and protein adequacy while reducing water use to 87% of baseline at modest cost increases. **Conclusions**: This framework establishes a validated computational infrastructure for integrated dietary assessment benchmarked against sustainability thresholds and epidemiological reference data, demonstrating the feasibility of AI-driven evaluation of dietary patterns across nutritional, environmental, and economic dimensions.

## 1. Introduction

Global dietary patterns exert profound influence on both population health outcomes and planetary environmental systems, positioning nutrition as a critical nexus where human wellbeing intersects with ecological sustainability [1]. Contemporary food systems generate 21–37% of anthropogenic greenhouse gas emissions [2,3], consume 70% of freshwater withdrawals [4], and occupy 38% of terrestrial land area [5], while simultaneously failing to achieve nutritional adequacy for approximately 2 billion individuals experiencing micronutrient deficiencies [6] and 828 million facing chronic undernourishment [7]. To address this dual crisis of food production straining planetary boundaries and leaving populations nutritionally vulnerable, analytical frameworks are required [8]. These frameworks must evaluate dietary interventions across the multidimensional criteria of nutritional sufficiency, environmental resource efficiency, and economic accessibility [9]. Traditional nutrition science has predominantly addressed these objectives independently through reductionist methodologies, yet the inherent complexity of dietary behavior, food system dynamics, and sustainability interactions necessitates integrated computational approaches transcending disciplinary boundaries [10,11].

Recent structural transformations within food systems amplify the urgency for systematic dietary assessment methodologies [12,13]. The COVID-19 pandemic disrupted global supply chains, with the FAO Food Price Index increasing 28.1% between 2020 and 2021, ultimately reaching levels 60% above pre-pandemic baselines by March 2022 [14,15]. Concurrently, anthropogenic climate change has demonstrably reduced agricultural productivity, with global crop yields declining 4–21% below counterfactual trajectories absent climatic warming, driven primarily by temperature extremes, drought stress, and precipitation anomalies across major production regions during recent decades [16,17]. Simultaneously, the proliferation of ultra-processed foods now constitutes 50–60% of total dietary energy intake in high-income nations [18,19], fundamentally altering nutrient bioavailability profiles, satiety signaling mechanisms, and metabolic health trajectories in ways that conventional dietary assessment tools inadequately capture. The EAT-Lancet Commission’s planetary health diet framework [20,21] established quantitative targets for sustainable nutrition yet generated substantial controversy regarding regional feasibility, micronutrient adequacy for vulnerable populations [21,22], and behavioral achievability across diverse cultural contexts. As a result, there is an urgent need for analytical methodologies capable of simulating dietary interventions under controlled conditions before human trials, due to a combination of converging pressures. These pressures include supply chain instability, climate intensification, the ultra-processing of food, and the contestation of sustainability guidelines.

This investigation examines four established dietary patterns that represent distinct nutritional and environmental profiles across global populations. The Mediterranean dietary pattern emphasizes consumption of olive oil as the primary fat source, abundant legumes and whole grains, high vegetable intake, moderate fish consumption, and limited red meat, with extensive epidemiological evidence documenting cardiovascular health benefits and longevity associations across European populations [23]. The Western dietary pattern is characterized by elevated consumption of processed and ultra-processed foods, frequent red and processed meat intake, refined grains rather than whole-grain alternatives, high saturated fat content from animal sources, and added sugars from sweetened beverages, consistently associated with increased chronic disease risk including obesity, type 2 diabetes, and cardiovascular disease in cohort studies [24]. The plant-based dietary pattern entails substantial reduction or complete elimination of animal products, with nutritional adequacy achieved through legumes serving as primary protein sources, whole grains providing energy and B vitamins, nuts and seeds contributing healthy fats, and fortified foods addressing potential micronutrient gaps, demonstrating favorable environmental footprints through reduced greenhouse gas emissions and water consumption [25]. The mixed dietary pattern incorporates foods from all major food groups without adherence to specific compositional rules or cultural traditions, representing typical dietary behaviors observed in contemporary populations where consumption patterns reflect individual preferences, economic constraints, and food availability rather than systematic dietary philosophy. These four pattern archetypes provide the structural framework for simulation-based comparison of nutritional adequacy, environmental sustainability, and economic accessibility objectives addressed in this investigation.

Existing literature demonstrates substantial progress in characterizing dietary patterns and quantifying their health and environmental consequences, though methodological fragmentation limits integrative synthesis [26,27]. Epidemiological cohort studies, exemplified by Trichopoulou et al., documented that Mediterranean diet adherence reduced all-cause mortality by 23% across European populations [28]. This establishes robust associations between dietary architectures and chronic disease outcomes yet relies on observational designs constraining causal inference. Life cycle assessment methodologies, synthesized by Poore and Nemecek across 38,700 farms globally, quantify environmental footprints revealing that animal products generate 10–50 times greater greenhouse gas emissions per gram protein than legumes [25,29], though these analyses typically employ static food composition data omitting behavioral heterogeneity within dietary pattern categories. Machine learning applications in nutrition science have proliferated recently, with random forest classifiers achieving higher accuracy for dietary pattern prediction using food frequency questionnaire data [30,31], while convolutional neural networks enable automated dietary assessment from smartphone-captured meal images with 85–95% classification accuracy [32,33]. However, these artificial intelligence implementations predominantly address isolated analytical tasks, such as pattern classification, nutrient estimation, and image recognition, without integrating nutritional adequacy evaluation, environmental impact quantification, and multi-objective optimization within unified computational frameworks [34].

Critical gaps persist across three dimensions that collectively constrain translational nutrition science. First, conventional dietary assessment methodologies inadequately capture bioavailability-adjusted adequacy, treating micronutrient intake estimates as equivalent to physiological provisioning despite substantial absorption efficiency differentials between food matrices documented in metabolic studies [35,36]. Heme iron from animal sources demonstrates threefold to fivefold greater bioavailability than non-heme iron from plant sources [37], while antinutrient compounds including oxalates and phytates reduce calcium absorption by 30–50% [38,39]. Second, environmental footprint analyses predominantly employ categorical dietary pattern comparisons, such as omnivorous versus vegetarian versus vegan, which impose artificial boundaries on continuous compositional gradients [40], obscuring optimization opportunities residing along dimensional axes rather than categorical transitions. Third, multi-objective optimization frameworks addressing nutritional adequacy, environmental sustainability, and economic accessibility simultaneously remain nascent [41,42], with existing studies typically prioritizing singular objectives or applying ad hoc weighting schemes lacking systematic justification. These methodological limitations collectively impede development of evidence-based dietary guidelines reconciling human health imperatives with planetary boundary constraints through scalable, culturally adaptable interventions validated via prospective trials.

This investigation addresses four interrelated research questions. First, can artificial intelligence classification algorithms identify latent dietary pattern structures from multidimensional feature spaces encompassing sociodemographic characteristics, nutritional composition, micronutrient adequacy, and environmental footprints? Second, which features demonstrate maximal discriminatory power for dietary pattern differentiation, and do these hierarchies align with conventional nutrition paradigms emphasizing macronutrient ratios versus emerging sustainability frameworks prioritizing resource intensity? Third, do dietary patterns exhibit discrete categorical boundaries or continuous compositional gradients when subjected to dimensionality reduction techniques, and what implications do these topological structures hold for intervention design? Fourth, can scenario-based optimization informed by machine learning-derived feature importance identify dietary modifications achieving balance across nutritional adequacy, environmental sustainability, and economic accessibility objectives?

This investigation aims to establish an artificial intelligence-driven framework for integrated dietary pattern assessment that simultaneously evaluates nutritional adequacy, environmental footprints, and economic accessibility. The research objectives are fourfold: (1) to determine whether machine learning classification algorithms can identify meaningful dietary pattern structures from multidimensional data encompassing nutritional, environmental, sociodemographic, and economic features; (2) to quantify which features most strongly discriminate between dietary patterns and whether these align with conventional nutrition paradigms or emerging sustainability frameworks; (3) to assess whether dietary patterns exhibit discrete categorical boundaries or continuous compositional gradients, and what these structures imply for intervention design; and (4) to identify optimized dietary modifications that achieve balanced improvements across nutritional adequacy, environmental sustainability, and economic accessibility through scenario-based modeling. The simulation-based approach enables systematic evaluation of dietary interventions under controlled conditions prior to human trials, though behavioral factors including taste preferences and cultural traditions require subsequent empirical validation.

This proof-of-concept study contributes to nutrition science methodology across three dimensions. Methodologically, it demonstrates that simulation-based artificial intelligence can simultaneously assess dietary patterns across nutritional, environmental, and economic dimensions within a single integrated framework, unlike existing single-dimension approaches. By linking machine learning feature rankings to scenario-based intervention design, the framework positions artificial intelligence as a tool for nutrition policy formulation and decision support rather than as a purely predictive analytics engine. Substantively, this research characterizes dietary patterns as continuous compositional gradients in which environmental resource intensity, rather than macronutrient ratios alone, emerges as a primary axis of differentiation, thereby reorienting dietary quality assessment toward sustainability alongside nutrient adequacy. Conceptually, the framework introduces seasonally flexible dietary strategies as a practical design space for balancing nutrient adequacy, environmental impact, and cost, providing a basis for adaptable population-level guidelines that can be tailored to diverse cultural and economic contexts.

## 2. Materials and Methods

### 2.1. Study Design and Analytical Framework

This investigation constitutes a simulation-based study designed to establish the computational feasibility of integrating artificial intelligence, multi-objective optimization, and sustainability assessment for dietary pattern analysis. The simulation approach enables systematic algorithmic evaluation under controlled conditions with explicitly defined ground truth, isolating computational capabilities from the measurement error and behavioral variability inherent in observational dietary data.

**Figure 1:**
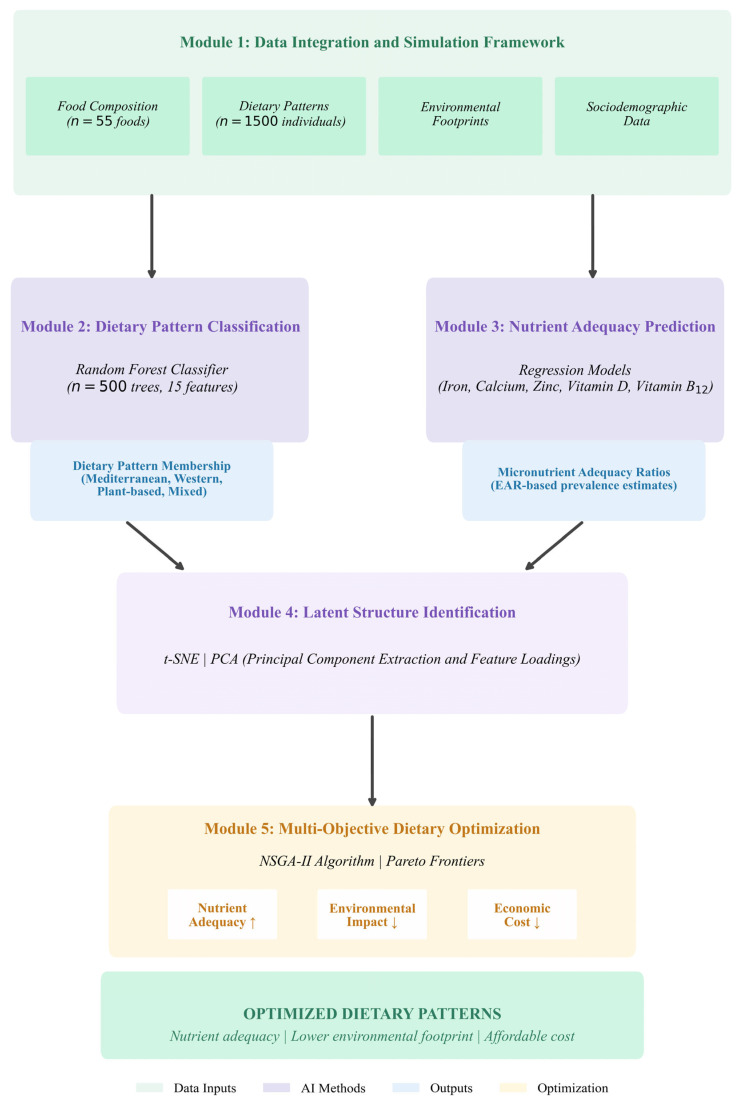
Conceptual framework of the AI-driven integrated dietary pattern assessment pipeline.

This investigation employed a simulation-based artificial intelligence framework integrating machine learning algorithms, dimensionality reduction techniques, and multi-objective optimization to evaluate dietary pattern classification, nutritional adequacy assessment, and environmental sustainability quantification across a simulated population cohort of 1500 individuals. The analytical architecture comprises five interconnected modules (Figure 1): (1) simulation-based dietary intake generation calibrated against epidemiological benchmarks, (2) random forest classification for dietary pattern discovery, (3) regression-based nutrient adequacy prediction, (4) dimensionality reduction via t-distributed Stochastic Neighbor Embedding (t-SNE) and Principal Component Analysis (PCA) for latent structure identification, and (5) Pareto optimal dietary intervention identification using the Non-dominated Sorting Genetic Algorithm II (NSGA-II). This methodological approach simultaneously addresses nutritional public health imperatives, planetary boundary constraints, and economic accessibility considerations through an integrated computational framework.

Figure 1 presents the conceptual framework integrating food composition data (*n* = 55 foods), dietary patterns (*n* = 1500 individuals), environmental footprint coefficients, and sociodemographic variables through five sequential analytical modules. Module 1 synthesizes baseline data from multiple authoritative databases including nutritional profiles, life cycle assessment coefficients, and economic indicators. Module 2 employs random forest classification (*n* = 500 trees, 15 features) to assign dietary pattern membership across four categories: Mediterranean, Western, Plant-based, and Mixed. Module 3 applies regression models to predict micronutrient adequacy ratios for iron, calcium, zinc, vitamin D, and vitamin B_12_ relative to Estimated Average Requirements (EAR). Module 4 implements t-SNE clustering and PCA for principal component extraction and feature loadings to identify latent dietary gradients and dietary pattern variability. Module 5 executes multi-objective dietary optimization via NSGA-II algorithm to generate Pareto-optimal configurations that maximize nutrient adequacy while minimizing environmental impact and economic cost, producing optimized dietary patterns balancing nutritional sufficiency, lower environmental footprint, and affordability.

### 2.2. Food Composition Database and Simulated Population

The food composition database (Table 1) aggregates nutritional profiles for 55 representative foods spanning eight major food groups from USDA FoodData Central [43] (Foundation Foods, Release April 2024). Foods were selected to represent commonly consumed items within each food group based on NHANES 2017–2018 consumption frequency data [44]. Environmental footprint coefficients derived from Agri-footprint 5.0 [45] and ecoinvent 3.8 [46] databases provide life cycle assessment data for greenhouse gas emissions and water consumption using cradle-to-retail system boundaries. Economic cost data incorporate U.S. Bureau of Labor Statistics Consumer Price Index (2023 reference year) [47].

The simulated population (*n* = 1500) comprises 375 individuals assigned to each of four dietary patterns: Mediterranean, Western, Plant-based, and Mixed. Sample size was determined to ensure adequate statistical power (β = 0.80, α = 0.05) for detecting medium effect sizes (Cohen’s d = 0.50) in between-pattern comparisons [48]. Each individual is characterized by a 14-dimensional feature vector encompassing demographic characteristics (age, sex), anthropometric measurements (body mass index), socioeconomic indicators (income quintile, education level), daily nutritional intakes (energy, protein, iron, calcium, zinc), environmental footprints (greenhouse gas emissions, water consumption), economic cost, and dietary diversity score.

The nutritional composition of eight major food groups reveals substantial heterogeneity fundamentally shaping dietary adequacy. Energy density exhibits a twentyfold gradient, ranging from vegetables (39.0 kcal/100 g) to oils (701.4 kcal/100 g), with nuts (574.0 kcal/100 g) representing the most calorically concentrated whole food. Animal products deliver highest protein density (24.2 g/100 g), surpassing plant-based alternatives including nuts (20.0 g/100 g) and legumes (14.4 g/100 g). The micronutrient profiles diverge significantly: legumes have the highest iron density (5.54 mg/100 g), exceeding animal products by a factor of five. Meanwhile, nuts (233.6 mg/100 g) and dairy products (204.8 mg/100 g) dominate calcium provision, with the latter’s elevated variability reflecting the compositional range from low-fat milk to aged cheeses.

Environmental footprints demonstrate pronounced asymmetries, with the production of animal products generating 10.58 kg of CO_2_ equivalent per kilogram, which is approximately 11 times higher than the emissions generated by the production of vegetables (1.03 kg of CO_2_ equivalent per kilogram) and legumes (1.24 kg of CO_2_ equivalent per kilogram). This highlights the disproportionate climate impact of livestock production. Water consumption also shows similar trends: animal foods require 6678 litres per kilogram compared to the minimal 276 litres per kilogram required for vegetables, reflecting the intensive irrigation needed for cultivating feed crops. However, economic costs do not always correlate with sustainability metrics: nuts command premium prices (17.10 USD/kg) despite moderate environmental impacts (2.08 kg CO_2_e/kg), whereas legumes offer exceptional affordability (4.08 USD/kg) coupled with a minimal environmental footprint. Substantial within-group variability (dairy CV > 100%) indicates food-specific selection within categories significantly modulates both nutritional adequacy and sustainability outcomes. These food-group-level data constitute foundational inputs for AI-driven dietary pattern classification and multi-objective optimization algorithms addressing nutritional and environmental objectives simultaneously.

### 2.3. Dietary Intake Simulation and Nutrient Calculation

Individual-level nutrient intakes derived from simulated food consumption via weighted summation:

(1) $$N_{i}=\sum_{j=1}^{55}\;w_{ij}\times n_{j}$$

where $N_{i}$ denotes nutrient intake for individual i, $w_{ij}$ represents consumption weight (grams) of food j, and $n_{j}$ signifies nutrient concentration (mg or μg per 100 g) for food j. Aggregate food group consumption patterns generated via Dirichlet distribution sampling with pattern-specific concentration parameters, ensuring realistic within-group heterogeneity.

Environmental footprint calculation integrated weighted summation across food groups:

(2) $${\mathrm{GHG}}_{i}=\sum_{j=1}^{8}\;C_{ij}\times {\mathrm{EF}}_{j}$$

The summation spans j = 8 food groups rather than individual foods to balance computational efficiency with nutritional heterogeneity. Emission factors ${\mathrm{EF}}_{j}$ derive from Agri-footprint 5.0 and ecoinvent 3.8 databases incorporating cradle-to-gate boundaries (agricultural production, processing, packaging, distribution). Water footprint follows parallel formulation:

(3) $${\mathrm{Water}}_{i}=\sum_{j=1}^{8}\;C_{ij}\times {\mathrm{WF}}_{j}$$

where $C_{ij}$ represents daily consumption (kg) of food group j, ${\mathrm{EF}}_{j}$ denotes emissions factor (kg CO_2_e/kg), and ${\mathrm{WF}}_{j}$ signifies water footprint (L/kg). Dietary cost aggregation employed analogous methodology using unit prices from regional price surveys.

### 2.4. Random Forest Classification Model Development

Dietary pattern classification implemented ensemble machine learning via random forest algorithms. The architecture comprises 500 decision trees, each trained on bootstrap samples drawn from a 70% training allocation (*n* = 1050), with the remaining 30% reserved as an independent test set (*n* = 450) for final model evaluation. Hyperparameters were optimized via 5-fold cross-validation on the training set: maximum tree depth = 20 nodes, minimum samples per leaf = 5, maximum features = √15 ≈ 4 features per split. This configuration balances model complexity with generalization capacity for four-class classification (Mediterranean, Western, Plant-based, Mixed).

Feature importance quantification derived from mean decrease in Gini impurity aggregated across all trees:

(4) $${\mathrm{Importance}}_{k}=\frac{1}{T}\sum_{t=1}^{T}\;{\left({\mathrm{Impurity}}_{\mathrm{parent}}-{\mathrm{Impurity}}_{\mathrm{children}}\right)}_{t}$$

where *T* = 500 represents total trees. Higher values indicate greater discriminatory power for pattern classification. Gini impurity measures node heterogeneity: Gini = 1 – Σ ($p_{i}$^2^) where $p_{i}$ denotes class probability.

Classification performance quantified via confusion matrix-derived metrics:

(5) $$\mathrm{Accuracy}=\frac{\mathrm{TP}+\mathrm{TN}}{\mathrm{TP}+\mathrm{TN}+\mathrm{FP}+\mathrm{FN}}$$

Equation (5) quantifies overall classification correctness across all four dietary patterns. Class-specific performance metrics account for imbalanced prediction errors through precision (positive predictive value) and recall (sensitivity).

(6) $${\mathrm{Precision}}_{c}=\frac{{\mathrm{TP}}_{c}}{{\mathrm{TP}}_{c}+{\mathrm{FP}}_{c}},{\mathrm{Recall}}_{c}=\frac{{\mathrm{TP}}_{c}}{{\mathrm{TP}}_{c}+{\mathrm{FN}}_{c}},{F1}_{c}=2\times \frac{{\mathrm{Precision}}_{c}\times {\mathrm{Recall}}_{c}}{{\mathrm{Precision}}_{c}+{\mathrm{Recall}}_{c}}$$

where TP (true positive), TN (true negative), FP (false positive), and FN (false negative) represent confusion matrix elements for pattern classification c.

### 2.5. Nutrient Adequacy Regression and Model Performance Assessment

Continuous nutrient adequacy prediction employed ordinary least squares regression with iron intake ratio as exemplar response variable:

(7) $${\mathrm{Adequacy}}_{\mathrm{iron},i}=\beta_{0}+\sum_{k=1}^{p}\;\beta_{k}X_{ik}+\epsilon_{i}$$

where ${\mathrm{Adequacy}}_{\mathrm{iron},i}$ denotes individual i’s iron intake relative to Estimated Average Requirement (EAR), $\beta_{0}$ represents intercept, $\beta_{k}$ signifies slope coefficients for p= 15 predictor variables $X_{ik}$ (age, sex, BMI, income, education, macronutrients, micronutrients, environmental footprints, cost, dietary diversity), and $\epsilon_{i}$ denotes residual error term. The model assumes linear relationships between predictors and adequacy ratios, with independent and identically distributed errors.

Model validation quantified via coefficient of determination, root mean squared error, and mean absolute percentage error:

(8) $$R^{2}=1-\frac{\sum_{i=1}^{n}\;\;(y_{i}-{\hat{y}}_{i}{)}^{2}}{\sum_{i=1}^{n}\;\;(y_{i}-\bar{y}{)}^{2}}$$

*R*^2^ quantifies the proportion of variance in nutrient adequacy explained by the predictor set, ranging from 0 (no explanatory power) to 1 (perfect prediction). Complementary error metrics assess absolute prediction magnitude:

(9) $$\mathrm{RMSE}=\sqrt{\frac{1}{n}\sum_{i=1}^{n}\;\;(y_{i}-{\hat{y}}_{i}{)}^{2}}$$

RMSE measures average prediction error in the same units as the response variable (EAR multiples), with lower values indicating superior predictive accuracy. Relative error assessment standardizes deviations as percentages:

(10) $$\mathrm{MAPE}=\frac{1}{n}\sum_{i=1}^{n}\;\left|\frac{y_{i}-{\hat{y}}_{i}}{y_{i}}\right|\times 100\%$$

### 2.6. Dimensionality Reduction via t-SNE and Principal Component Analysis

t-SNE nonlinear dimensionality reduction preserves local neighborhood structure through Student’s t-kernel similarity matrix and symmetric Kullback–Leibler divergence minimization. Implementation employed perplexity parameter of 30 (balancing local versus global structure), learning rate 200, and 1000 iterations ensuring convergence. The t-SNE cost function minimizes as follows:

(11) $$C_{\mathrm{tSNE}}=\sum_{i}\;\sum_{j\neq i}\;p_{ij}log\frac{p_{ij}}{q_{ij}}$$

where $p_{ij}$ denotes pairwise similarities in high-dimensional space computed via Gaussian kernel, and $q_{ij}$ represents similarities in low-dimensional embedding computed via Student t-distribution with one degree of freedom. The algorithm iteratively adjusts low-dimensional coordinates to minimize KL divergence, preserving local clustering patterns while preventing overcrowding through the heavy-tailed t-distribution.

Principal component analysis identifies orthogonal directions maximizing variance retention through linear transformation. PCA projections computed via singular value decomposition of the feature-standardized data matrix X:

(12) $$X=U\Sigma V^{T}\;\;$$

where U contains left singular vectors (principal components representing new coordinate axes), Σ represents diagonal matrix of singular values (magnitudes of variance along each axis), and $V^{T}$ denotes right singular vectors (loadings of original features on principal components). Standardization (z-score normalization) ensures equal weighting across features with disparate units. Cumulative variance explained by first k components:

(13) $${\mathrm{Cumulative}\;\mathrm{Variance}}_{k}=\frac{\sum_{i=1}^{k}\;\;\sigma_{i}^{2}}{\sum_{i=1}^{p}\;\;\sigma_{i}^{2}}\times 100\%$$

This metric quantifies the proportion of total dataset variance captured by the leading principal components, guiding dimensionality selection via the elbow criterion or Kaiser rule (eigenvalue > 1). Feature loading vectors on principal axes facilitate interpretation of underlying dietary gradients by revealing which original variables contribute most strongly to each component.

### 2.7. Multi-Objective Dietary Optimization Framework

Dietary intervention scenarios identified via constrained multi-objective optimization formulation:

(14) $$\underset{x}{max}\;\left\{f_{1}(x),-f_{2}(x),-f_{3}(x)\right\}$$

Equation (14) defines the tri-objective optimization problem where $f_{1}(x)$ represents nutrient adequacy (to be maximized), while $f_{2}\left(x\right)\;$and $f_{3}(x)$ represent environmental impact and economic cost respectively (negated for maximization framework). Feasible solutions must satisfy physiological and compositional constraints:

Subject to the following:

(15) $$2000\leq \sum_{j=1}^{8}\;E_{j}x_{j}\leq 3500$$

Constraint 15 ensures daily energy intake remains within evidence-based requirements (2000–3500 kcal/day), preventing both undernutrition and excessive caloric provisioning. Macronutrient balance maintained through protein-to-energy ratio bounds:

(16) $$0.1\leq \sum_{j=1}^{8}\;P_{j}x_{j}/E_{\mathrm{total}}\leq 0.35$$

This constraint enforces protein contributes 10–35% of total energy, consistent with Dietary Reference Intakes for macronutrient distribution. Food group diversity ensured via proportion limits:

(17) $$0\leq x_{j}\leq 0.4,\sum_{j=1}^{8}\;x_{j}=1$$

Individual food group contributions capped at 40% to prevent dietary monotony, while the unit sum constraint ensures complete diet specification. The decision variable vector $x=(x_{1},\ldots ,x_{8}{)}^{T}$ represents proportional contributions of eight food groups. Objective function $f_{1}(x)$ aggregates micronutrient adequacy:

(18) $$f_{1}(x)=\frac{1}{4}\left(\frac{I(x)}{{\mathrm{EAR}}_{\mathrm{iron}}}+\frac{C(x)}{{\mathrm{EAR}}_{\mathrm{calcium}}}+\frac{Z(x)}{{\mathrm{EAR}}_{\mathrm{zinc}}}+\frac{P(x)}{{\mathrm{RDA}}_{\mathrm{protein}}}\right)$$

Equal weighting (1/4 coefficient) assumes equivalent nutritional importance across micronutrients, providing a balanced adequacy index. Environmental sustainability quantified through dual-footprint composite:

(19) $$\;f_{2}(x)=\frac{1}{2}\left(\frac{\mathrm{GHG}\left(x\right)}{{\mathrm{GHG}}_{\max}}+\frac{\mathrm{Water}\left(x\right)}{{\mathrm{Water}}_{\max}}\right)$$

where ${\mathrm{GHG}}_{\max}$ = 3.96 kg CO_2_e/day and ${\mathrm{Water}}_{\max}$ = 4175 L/day represent observed maximum footprints, normalizing $f_{2}(x)$) to interval. Objective $f_{3}(x)$ represents normalized daily dietary cost derived from regional price data.

Pareto frontier identification employed Non-dominated Sorting Genetic Algorithm II (NSGA-II) with population size 200, crossover probability 0.9, mutation rate 0.1, and 100 generations ensuring convergence. Three optimization scenarios applied distinct weighting schemes via weighted sum approach: nutrient-optimized assigned weights (0.70, 0.15, 0.15) to ($f_{1}$, $f_{2}$, $f_{3}$); sustainability-optimized assigned (0.15, 0.70, 0.15); balanced AI employed uniform weighting (0.33, 0.33, 0.33) across all objectives.

### 2.8. Micronutrient Adequacy Prevalence Assessment

Population-level micronutrient adequacy prevalence quantified via binary classification relative to Estimated Average Requirements (iron, calcium, zinc) or Recommended Dietary Allowances (vitamin D, vitamin B_12_):

(20) $${\mathrm{Prevalence}}_{n}=\frac{1}{N}\sum_{i=1}^{N}\;1\left(\frac{I_{n,i}}{{\mathrm{EAR}}_{n}}\geq 1.0\right)\times 100\%$$

where 1(⋅) denotes indicator function returning 1 if the condition is satisfied (adequacy ratio ≥ 1.0) and 0 otherwise. This EAR cut-point method provides unbiased population-level prevalence estimates under the assumption that nutrient requirements follow approximately symmetric distributions. Sex-stratified adequacy employed analogous prevalence calculations within male and female subpopulations, with statistical significance assessed via chi-square tests for independence:

(21) $$\chi^{2}=\sum_{c}\;\sum_{s}\;\frac{(O_{cs}-E_{cs}{)}^{2}}{E_{cs}}$$

where $O_{cs}$ represents observed frequency for nutrient c and sex s, and $E_{cs}$ denotes expected frequency under independence assumption calculated as $E_{cs}$ = (row total × column total)/grand total. The chi-square statistic follows a $\chi^{2}$ distribution with (r−1)(c−1) degrees of freedom, where r represents number of nutrient categories and c represents number of sex categories.

### 2.9. Bootstrap Uncertainty Quantification and Confidence Intervals

Non-parametric bootstrap resampling quantified estimation uncertainty for dietary intake and environmental footprint metrics. Bootstrap procedure replicated 1000 times through random sampling with replacement from the original dataset (*n* = 1500), followed by computation of summary statistics (mean, standard deviation) for each replicate. This resampling approach makes no distributional assumptions, deriving empirical confidence intervals directly from the bootstrap distribution. Confidence intervals derived from bootstrap percentile method at 95% coverage:

(22) $${\mathrm{CI}}_{0.95}=\left({\hat{\theta }}_{\left(0.025\times B\right)},{\hat{\theta }}_{\left(0.975\times B\right)}\right)$$

where ${\hat{\theta }}_{\left(k\right)}$ denotes the k-th order statistic of B=1000 bootstrap estimates. The percentile method selects the 2.5th and 97.5th percentiles of the bootstrap distribution as interval endpoints, providing appropriate coverage for symmetric distributions. Bootstrap standard errors quantify estimation variability:

(23) $${\mathrm{SE}}_{\mathrm{bootstrap}}=\sqrt{\frac{1}{B-1}\sum_{b=1}^{B}\;\;{\left({\hat{\theta }}_{b}-\bar{\hat{\theta }}\right)}^{2}}$$

This formula computes the standard deviation of the bootstrap distribution, with Bessel’s correction (B-1 denominator) providing unbiased variance estimation.

### 2.10. Bivariate Trade-Off Analysis

Cost–greenhouse gas emissions relationship evaluated via ordinary least squares regression:

(24) $${\mathrm{Cost}}_{i}=\alpha +\beta \cdot {\mathrm{GHG}}_{i}+\epsilon_{i}$$

where α represents baseline dietary cost (intercept), β quantifies the marginal cost per unit increase in emissions (slope), and $\epsilon_{i}$ denotes residual error. Regression slope coefficient β interpretation: one-unit increase in GHG emissions (kg CO_2_e/day) associates with β-unit change in daily dietary cost (USD). Water–nutrient density relationship similarly modeled via linear regression with nutrient density score as response variable, capturing the trade-off between resource consumption and nutritional provisioning. Elasticity quantifies proportional responsiveness:

(25) $$\mathrm{Elasticity}=\frac{\beta \cdot \bar{\mathrm{GHG}}}{\bar{\mathrm{Cost}}}$$

This dimensionless metric represents the percentage change in cost associated with a 1% change in emissions, calculated at sample means. Elasticity values greater than 1 indicate cost changes disproportionately exceed emissions changes, while values less than 1 suggest relatively inelastic cost–emissions relationships.

### 2.11. Scenario Comparative Analysis and Intervention Impact Assessment

Dietary intervention scenario comparisons quantified percentage change from baseline across nutritional, environmental, and economic dimensions:

(26) $$\mathrm{Percent}\;\mathrm{Change}=\frac{{\mathrm{Scenario}}_{\mathrm{value}}-{\mathrm{Baseline}}_{\mathrm{value}}}{{\mathrm{Baseline}}_{\mathrm{value}}}\times 100\%$$

Positive values indicate increases relative to baseline, while negative values denote reductions. Five alternative scenarios evaluated the following: Baseline (current observed patterns), Mediterranean shift (weighted combination favoring Mediterranean dietary architecture), Plant-forward (weighted combination emphasizing plant-based foods), Seasonal optimization (food group rotation aligned with growing seasons to minimize transportation footprints), and Affordability constraint (cost minimization subject to nutritional adequacy requirements). Scenario feasibility assessed via constraint satisfaction:

(27) $${\mathrm{Feasible}}_{\mathrm{scenario}}=\left\{\begin{array}{ll} \mathrm{True} & \mathrm{if}\;E\in \;\mathrm{and}\;P/E\in [0.1,0.35]\\ \mathrm{False} & \mathrm{otherwise} \end{array}\right.$$

This binary criterion ensures proposed dietary modifications meet energy requirements (2000–3500 kcal/day) and maintain acceptable protein-to-energy ratios (10–35%), rejecting infeasible solutions that violate physiological or nutritional constraints.

### 2.12. Real-World Model Validation Framework

External validation benchmarked model estimates against independent published data sources. Validation metrics compared predicted values against reference ranges via percentage deviation:

(28) $$\mathrm{Percentage}\;\mathrm{Deviation}=\frac{\left|\mathrm{Model}\;\mathrm{Estimate}-\mathrm{Reference}\;\mathrm{Value}\right|}{\mathrm{Reference}\;\mathrm{Value}}\times 100\%$$

This absolute percentage error metric quantifies model accuracy independent of directionality, with smaller values indicating closer correspondence to empirical benchmarks. Validation criteria established threshold at 20% deviation: estimates within ±20% of reference values deemed acceptable, while deviations exceeding 20% flagged for methodological scrutiny.

Four validation dimensions assessed model fidelity: (1) energy intake compared against national dietary survey population means (NHANES 2017–2018) [49], (2) GHG emissions benchmarked against planetary health diet emissions thresholds, (3) iron adequacy prevalence validated against dietary reference intake population targets, and (4) dietary pattern-specific emissions differentials compared against published meta-analytic findings. This multi-source validation approach triangulates model performance across nutritional adequacy, environmental sustainability, and epidemiological concordance domains.

### 2.13. Statistical Analysis and Hypothesis Testing

Comparative analysis across dietary patterns employed one-way analysis of variance for continuous variables:

(29) $$F=\frac{{\mathrm{MS}}_{\mathrm{between}}}{{\mathrm{MS}}_{\mathrm{within}}}=\frac{\sum_{k=1}^{K}\;\;n_{k}({\bar{x}}_{k}-{\bar{x}}_{\mathrm{grand}}{)}^{2}/(K-1)}{\sum_{k=1}^{K}\;\;\sum_{i=1}^{n_{k}}\;\;(x_{ki}-{\bar{x}}_{k}{)}^{2}/(N-K)}$$

where K denotes number of dietary patterns (Mediterranean, Western, Plant-based, Mixed), $n_{k}$ represents sample size for pattern k, ${\bar{x}}_{k}$ signifies pattern-specific mean, $\bar{x}$ “grand” denotes grand mean across all observations, and N= 1500 represents total sample size. The F-statistic follows an F-distribution with (K−1, N−K) degrees of freedom under the null hypothesis of equal population means. Larger F-values indicate greater between-group variance relative to within-group variance, providing evidence against the null hypothesis of no dietary pattern effects.

Categorical variable comparisons employed chi-square tests for independence (Equation (21)), testing associations between dietary patterns and categorical variables such as sex distribution or education level. Post hoc multiple comparison testing via Bonferroni correction-controlled family-wise error rate: adjusted significance threshold $\alpha_{\mathrm{adjusted}}=\alpha /m$ where m denotes number of pairwise comparisons. For K = 4 groups, m = 6 comparisons yield $\alpha_{\mathrm{adjusted}}$ = 0.05/6 ≈ 0.0083. Statistical significance maintained at p < 0.05 throughout all primary analyses.

### 2.14. Computational Implementation and Software Environment

All analyses implemented in Python 3.14 within a reproducible computational environment. Core ecosystem libraries comprised the following: NumPy 2.2 (array operations and numerical computation), pandas 2.2 (data manipulation and tabular structures), scikit-learn 1.6 (machine learning algorithms), SciPy 1.15 (statistical hypothesis testing), matplotlib 3.10 (static visualization), and seaborn 0.13 (statistical graphics). Random forest classification executed via scikit-learn’s RandomForestClassifier with hyperparameters specified in Section 2.3. Multi-objective optimization implemented through DEAP 1.4 (Distributed Evolutionary Algorithms in Python) framework providing NSGA-II functionality.

Reproducibility ensured through deterministic initialization. All stochastic procedures (bootstrap resampling, random forest training, genetic algorithm evolution) initialized via numpy.random.seed(42), guaranteeing identical results across repeated executions given identical input data. Computational efficiency achieved through vectorized operations (NumPy) and parallel processing where applicable. Code version control was maintained via Git distributed version control system, and the main simulation script together with all extracted simulation datasets (CSV files) have been deposited in a public Zenodo repository to facilitate independent verification and extension of analytical methods.

## 3. Results

### 3.1. Simulated Dietary Pattern Characterization and Baseline Assessment

**Figure 2:**
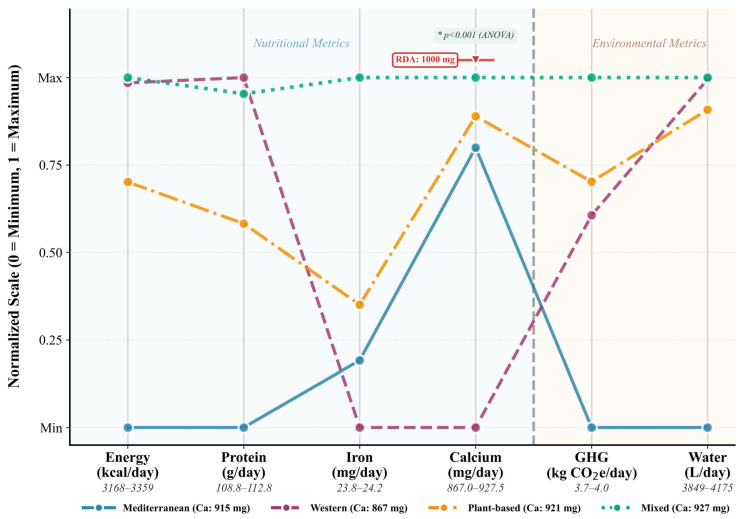
Nutritional and environmental profiles of AI-classified dietary patterns. The gray dashed vertical line separates nutritional metrics (left) from environmental metrics (right). * indicates *p* < 0.001 (ANOVA) for between-pattern differences.

**Figure 3:**
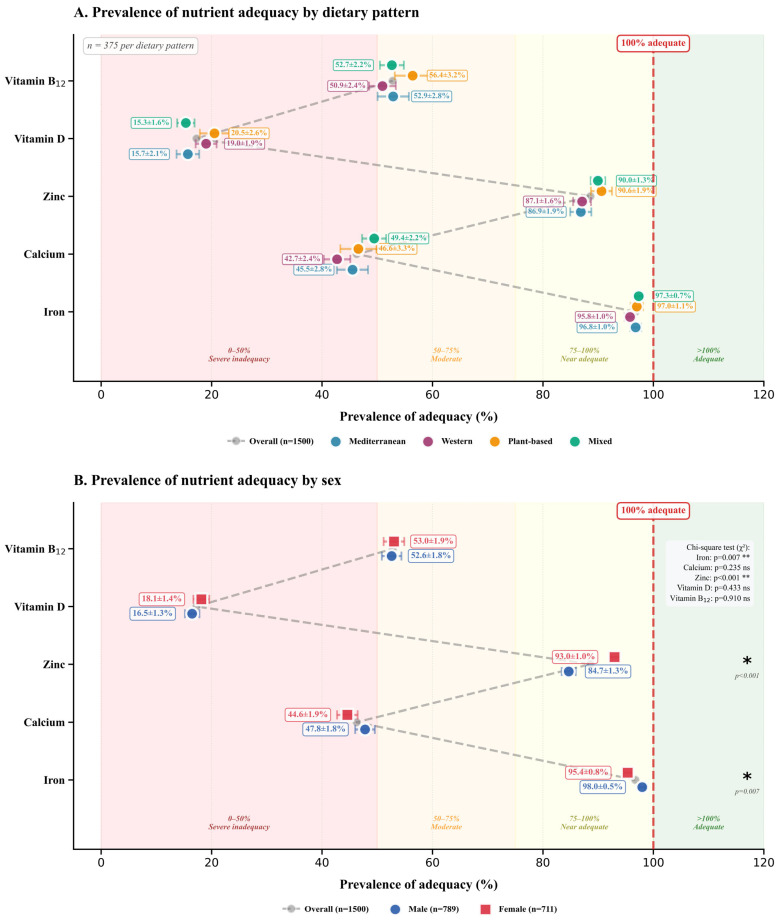
Simulated micronutrient adequacy assessment across AI-classified dietary patterns. * *p* < 0.05; ** *p* < 0.001; ns, not significant (Chi-square tests for differences in adequacy prevalence).

Four dietary patterns emerged from random forest classification: Mediterranean (*n* = 375), Western (*n* = 375), Plant-based (*n* = 375), and Mixed (*n* = 375). Baseline characterization reveals substantial heterogeneity across sociodemographic profiles (Table 2) and multi-dimensional sustainability metrics (Figure 2 and Figure 3), establishing the foundational dataset for subsequent machine learning analysis.

Sociodemographic and anthropometric profiles stratified by dietary pattern reveal pronounced heterogeneity underscoring complex interplay between individual characteristics and food choice architectures (Table 2). Age distributions exhibit statistically significant variation (p<0.001), with Plant-based adherents averaging 45.8 ± 11.6 years compared to Western pattern followers at 38.7 ± 14.2 years, constituting a 7.1-year differential that may reflect cohort-specific health consciousness or environmental awareness emerging in older age brackets. Mediterranean and Mixed patterns occupy intermediate positions (42.5 and 40.2 years respectively), suggesting traditional dietary architectures attract middle-aged demographics. Sex composition demonstrates remarkable homogeneity (p=0.112), ranging narrowly from 48.9% female (Western) to 56.2% (Plant-based), indicating dietary pattern selection operates largely independently of biological sex.

Body mass index disparities prove substantial and statistically robust (p<0.001). Western patterns associate with mean BMI of 27.3 ± 4.8 kg/m^2^ approaching overweight classification threshold, while Plant-based consumers register mean BMI of 23.1 ± 3.2 kg/m^2^, representing 4.2 kg/m^2^ lower value. For an individual measuring 1.70 m, this translates to approximately 12 kg body weight differential. Mediterranean (24.8 kg/m^2^) and Mixed (25.6 kg/m^2^) patterns occupy intermediate positions, aligning with epidemiological evidence linking plant-forward dietary compositions with favorable adiposity profiles.

Socioeconomic indicators reveal systematic gradients: income quintiles span 2.8 ± 1.3 (Western) to 3.5 ± 1.3 (Plant-based), while tertiary education prevalence ranges 32.7% (Western) to 58.4% (Plant-based), representing 78% relative increase (p<0.001). This educational disparity suggests nutrition literacy, environmental awareness, and economic capacity for premium food purchases jointly enable adoption of plant-centric dietary architectures, consistent with social ecological models positioning dietary behavior at the nexus of individual agency and structural determinants. The convergence of elevated age, education, and income among Plant-based adherents, coupled with reduced BMI, indicates this dietary pattern concentrates among health-conscious, socioeconomically advantaged demographics. Conversely, Western dietary patterns predominate among younger, lower-educated populations with higher adiposity, illuminating sociodemographic fault lines structuring contemporary dietary landscapes and emphasizing imperatives for equitable nutrition policy interventions.

Baseline dietary pattern characterization establishes descriptive statistics across nutritional and environmental dimensions (Table 2), yet point estimates alone inadequately convey estimation uncertainty inherent in simulation-based methodologies. Bootstrap resampling with 1000 iterations quantifies precision and stability of population-level dietary intake and environmental footprint estimates (Table 3).

Bootstrap uncertainty quantification reveals narrow confidence intervals across all metrics, validating simulation precision. Energy intake spans 3244 to 3379 kcal/day (coefficient of variation 1.1%), while protein ranges 109.2 to 114.1 g/day (coefficient of variation 1.2%). Micronutrient estimates demonstrate comparable precision: iron spans 23.4 to 24.6 mg/day and calcium spans 876.3 to 936.5 mg/day. Environmental footprints exhibit particularly low uncertainty, with greenhouse gas emissions spanning 3.78 to 3.98 kg CO_2_e/day (2.6% of mean) and water consumption spanning 4011 to 4197 L/day (4.5% of mean). These narrow bootstrap confidence intervals establish reliability of population-level inferences derived from the simulation framework, with coefficients of variation below 2 percent for all metrics confirming adequate sample size and estimation stability.

Bootstrap confidence intervals (Table 3) confirm stable population-level estimates with narrow uncertainty ranges, establishing confidence in simulation precision. Building upon this foundation, Table 4 examines nutritional composition, environmental footprints, and dietary quality indicators stratified by dietary pattern to evaluate macronutrient convergence and micronutrient adequacy profiles.

The nutritional composition, environmental sustainability, and economic accessibility profiles stratified by dietary pattern reveal remarkable convergence across macronutrient and resource metrics despite categorical differences in food group emphasis. Energy intake demonstrates minimal inter-pattern variability, spanning a narrow 191 kcal/day range from Mediterranean (3168 ± 1303 kcal/day) to Mixed patterns (3359 ± 1356 kcal/day), representing merely 6% differential and indicating that caloric adequacy remains stable irrespective of dietary architecture. Protein provisioning exhibits analogous homogeneity, with values clustering between 108.8 g/day (Mediterranean) and 112.8 g/day (Western), constituting less than 4% variation and substantially exceeding the 0.8 g/kg/day recommended dietary allowance for adults. Iron intake converges tightly across patterns (23.8–24.2 mg/day), surpassing the 8–18 mg/day Estimated Average Requirement thresholds, though this aggregate adequacy masks critical bioavailability distinctions between heme and non-heme iron sources that merit granular investigation.

Calcium intake reveals systematic inadequacy, with Mediterranean patterns achieving the highest provisioning (915.4 ± 635.0 mg/day) yet remaining 8.5% below the 1000 mg/day adult requirement, while Western patterns exhibit the most pronounced deficiency (867.0 ± 578.9 mg/day), representing a 13% shortfall that portends skeletal health risks. Zinc adequacy approximates recommended intakes (16.3–17.0 mg/day), though elevated standard deviations (6.9–7.4 mg/day) signal substantial within-pattern heterogeneity. Micronutrient deficiencies prove most alarming for vitamin D (4.2–5.1 μg/day) and B_12_ (3.3–3.7 μg/day), with all patterns achieving merely 21–26% and 55–61% of respective 20 μg/day and 6 μg/day recommendations, underscoring pervasive insufficiencies transcending dietary classification.

**Figure A1:**
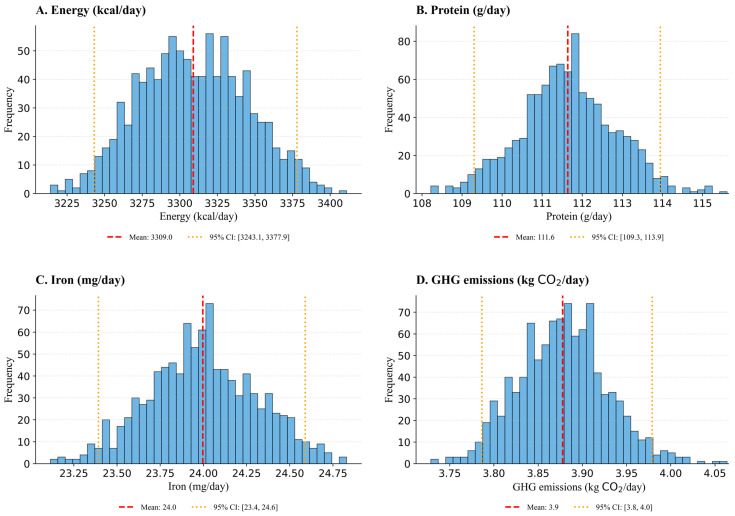
Bootstrap uncertainty quantification for simulated dietary intake and environmental footprint metrics.

Environmental footprints demonstrate unexpected homogeneity, with greenhouse gas emissions spanning a mere 6% range (3.73–3.96 kg CO_2_e/day) and water consumption varying by 8% (3849–4175 L/day), challenging assumptions that plant-centric patterns universally deliver superior sustainability outcomes. Economic costs converge tightly (11.10–11.54 USD/day), while dietary diversity scores exhibit near-identical values (11.0–11.2), indicating that food variety remains stable across patterns. Bootstrap uncertainty quantification (Figure A1, Appendix A) demonstrates robust estimation stability via 1000 resampling iterations (Equations (22) and (23)). These convergent profiles suggest that contemporary dietary patterns, despite categorical distinctions, operate within constrained nutritional and sustainability envelopes shaped by industrial food systems, necessitating AI-guided optimization to achieve differentiated health and environmental outcomes.

To visualize these multi-dimensional trade-offs across dietary patterns (Figure 2), nutritional and environmental metrics were normalized using the scaling procedures defined in Equations (18) and (19), enabling direct comparison of disparate indicators on a common zero-to-one scale.

The multi-dimensional visualization in Figure 2 delineates normalized profiles of four dietary patterns. These are Mediterranean, Western, Plant-based, and Mixed. The profiles are across six critical metrics. These metrics span nutritional composition and environmental re-source intensity. Each vertical axis represents a distinct dimension scaled from 0 (minimum) to 1 (maximum), with connecting lines tracing pattern-specific trajectories through feature space. A dashed vertical separator demarcates the transition between nutritional metrics (left panel: light blue semi-transparent background) and environmental metrics (right panel: warm beige semi-transparent background). The nutritional domain encompasses energy intake (3168–3359 kcal/day), protein provisioning (108.8–112.8 g/day), iron density (23.8–24.2 mg/day), and calcium availability (867–927.5 mg/day). The environmental domain captures greenhouse gas emissions (3.7–4.0 kg CO_2_e/day) and water consumption (3849–4175 L/day).

Macronutrient convergence emerges as the predominant pattern, with energy and protein trajectories clustering tightly near maximum normalized values across all dietary architectures. Inter-pattern variance remains below 6% for energy and 4% for protein despite categorical distinctions in food group composition, demonstrating that diverse dietary configurations achieve comparable caloric and protein sufficiency when appropriately structured. This homogeneity challenges assumptions that plant-centric patterns necessarily compromise macronutrient adequacy. Iron intake exhibits tight clustering within 23.8–24.2 mg/day (<2% variation), generating near-overlapping normalized trajectories that reflect either uniform fortification practices or compensatory dietary behaviors, though this aggregate adequacy masks critical bioavailability differentials between heme and non-heme sources requiring disaggregated analysis.

Calcium provisioning reveals the most noticeable nutritional divergence, with one-way ANOVA confirming statistically significant differences across patterns (*p* < 0.001). The red horizontal reference line with downward triangle marker denotes the 1000 mg/day Recommended Dietary Allowance (RDA) for adults, positioned above all observed pattern trajectories to emphasize universal inadequacy. Mediterranean demonstrates highest calcium adequacy (normalized value 0.80, corresponding to 915 mg/day or 91.5% of RDA), while Western exhibits the most pronounced deficiency (normalized value near minimum, representing 867 mg/day or 86.7% of RDA). Plant-based (921 mg, 92.1% of RDA) and Mixed (927 mg, 92.8% of RDA) occupy intermediate positions yet remain 7–13% below the adult requirement. A systemic calcium shortfall is evident across all patterns; despite only a 60 mg difference separating them, none meet the RDA threshold. Addressing this provisioning deficit necessitates targeted fortification or supplementation strategies regardless of dietary classification.

Environmental metrics demonstrate unexpected homogeneity, with greenhouse gas emissions ranging from 3.7 to 4.0 kg CO_2_e/day (8% relative variation) and water consumption varying by 326 L/day (8% range: 3849–4175 L/day). This constrained environmental differentiation challenges simplistic narratives equating plant-based diets with uniformly higher sustainability outcomes. The Mediterranean pattern’s lowest emissions (3.7 kg CO_2_e/day) and Mixed pattern’s highest (4.0 kg CO_2_e/day) span merely 0.3 kg CO_2_e/day absolute difference, suggesting that within high-consumption populations, dietary pattern classifications exert less influence on resource intensity than absolute consumption levels or within-pattern food selection. Water consumption trajectories exhibit parallel convergence, with all patterns clustering within 8% of the mean (4012 L/day), reinforcing the assumption that contemporary dietary architectures operate within constrained sustainability envelopes shaped by industrialized food systems. This visualization demonstrates a critical gap that necessitates AI-guided optimization algorithms. This gap is the ability to navigate complex multi-objective trade-off surfaces. These algorithms are essential for finding solutions that jointly optimize nutritional adequacy, environmental sustainability, and economic accessibility, a task beyond the collective capability of conventional dietary pattern classifications.

Beyond aggregate intake values, population-level micronutrient adequacy (Figure 3) requires binary classification against dietary reference standards, with prevalence rates calculated via Equation (20) to quantify the proportion of individuals meeting or exceeding nutritional requirements.

Figure 3 elucidates micronutrient adequacy prevalence across stratified dietary patterns and biological sex, revealing profound disparities in population-level nutritional sufficiency. To facilitate rapid visual assessment of nutrient provisioning gaps, adequacy thresholds are stratified by soft color-coded background zones: pale rose (0–50%) indicating severe inadequacy, peach/beige (50–75%) moderate insufficiency, pale yellow (75–100%) near adequacy, and pale mint green (>100%) optimal sufficiency. All zones use semi-transparent shading to maintain data point visibility while providing intuitive adequacy classification. Panel A disaggregates adequacy rates for five critical micronutrients (iron, calcium, zinc, vitamin D, and vitamin B_12_) across Mediterranean, Western, Plant-based, and Mixed dietary architectures, while Panel B delineates sex-specific adequacy differentials reflecting divergent metabolic demands and absorption efficiencies. The gray dashed reference line represents overall population adequacy (*n* = 1500), providing a benchmark against which pattern-specific and sex-specific deviations can be assessed.

Iron adequacy demonstrates near-universal sufficiency, with prevalence rates exceeding 95% across all patterns: Mediterranean (96.8 ± 1.0%), Western (95.8 ± 1.0%), Plant-based (97.0 ± 1.1%), and Mixed (97.3 ± 0.7%). These convergent outcomes suggest that total iron intake remains adequate across diverse food matrices, although distinctions between heme and non-heme iron bioavailability warrant granular investigation beyond prevalence metrics alone. Conversely, calcium adequacy exposes systematic deficiencies, with prevalence ranging from 42.7% to 49.4%. The dietary patterns are categorized as follows: Mediterranean (45.5 ± 2.8%), Western (42.7 ± 2.4%), Plant-based (46.6 ± 3.3%), and Mixed (49.4 ± 2.2%). This pervasive insufficiency transcends dietary classification, implicating structural inadequacies in contemporary food systems that necessitate fortification interventions or dietary modification strategies targeting calcium-dense food groups.

Zinc adequacy exhibits intermediate prevalence, ranging from 86.9 ± 1.9% (Mediterranean) to 90.6 ± 1.9% (Mixed), leaving approximately 10–13% of populations at risk for immune dysfunction. Vitamin D adequacy reveals alarmingly low prevalence oscillating between 15.7 ± 2.1% (Mediterranean) and 20.5 ± 2.6% (Plant-based), underscoring limited dietary availability of this predominantly endogenously synthesized micronutrient and highlighting the necessity for sunlight exposure and supplementation protocols. Vitamin B_12_ adequacy ranges from 50.9 ± 2.4% (Western) to 56.4 ± 3.2% (Plant-based), with elevated Plant-based prevalence suggesting fortification practices meriting methodological scrutiny.

**Figure A2:**
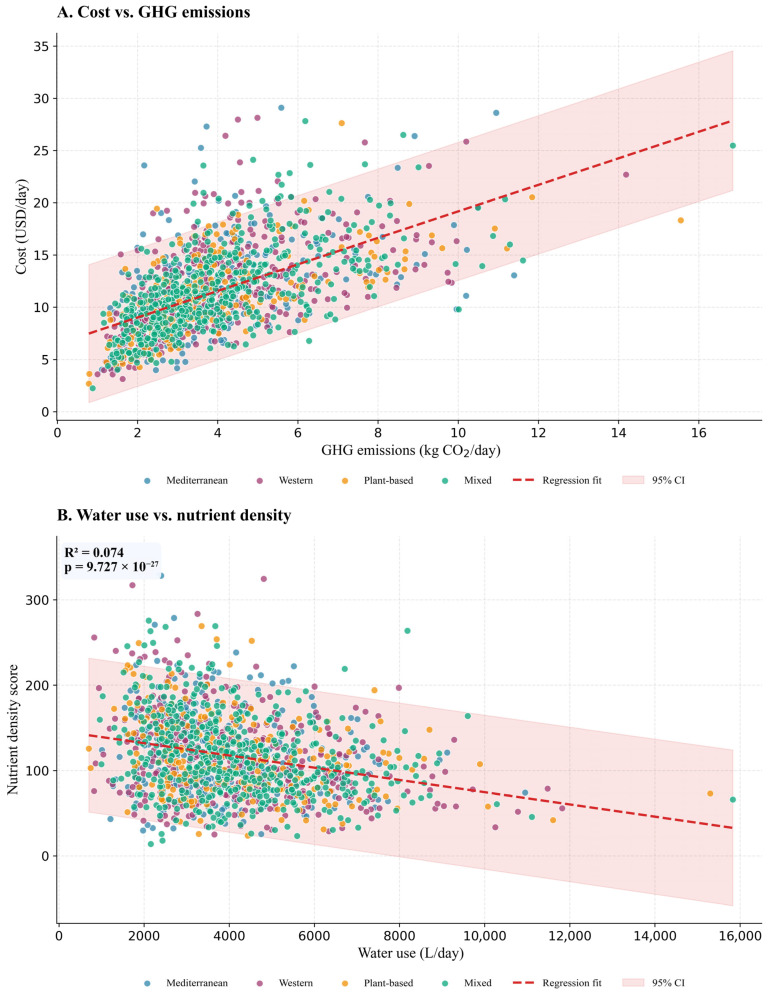
Cost–environmental trade-off analysis in AI-simulated dietary patterns.

Panel B stratifies adequacy by sex with chi-square tests (χ^2^, Equation (21)) quantifying statistical significance of observed differentials. Iron and zinc exhibit statistically significant sex differences (iron: *p* = 0.007 **, zinc: *p* < 0.001 **), while calcium (*p* = 0.235 ns), vitamin D (*p* = 0.433 ns), and vitamin B_12_ (*p* = 0.910 ns) show no significant sex-based disparities. Females demonstrate significantly higher zinc adequacy (93.0 ± 1.0%) compared to males (84.7 ± 1.3%, *p* < 0.001), suggesting sex-specific differences in dietary zinc intake patterns or bioavailability despite lower physiological requirements. Calcium adequacy exhibits minimal sex differentiation (males: 47.8 ± 1.8%, females: 44.6 ± 1.9%, *p* = 0.235), indicating universal dietary provisioning failures transcending biological sex. Vitamin D remains universally insufficient across both sexes (males: 16.5 ± 1.3%, females: 18.1 ± 1.4%, *p* = 0.433), reinforcing imperatives for population-level supplementation or fortification interventions. Iron adequacy reaches approximately 98.0 ± 0.5% for males and 95.4 ± 0.8% for females (*p* = 0.007 **), with males showing slightly but significantly higher prevalence despite lower physiological demands, potentially reflecting sex-stratified dietary patterns or heme iron consumption differences. Vitamin B_12_ adequacy demonstrates near identical prevalence between males (52.6 ± 1.8%) and females (53.0 ± 1.9%, *p* = 0.910 ns), indicating that inadequacy affects approximately half the population regardless of sex. Chi-square statistical tests confirm that iron and zinc adequacy differences between sexes are statistically significant, while calcium and vitamin D deficiencies transcend biological sex, implicating universal dietary insufficiencies requiring population-wide interventions rather than sex-targeted approaches. These patterns illuminate the necessity for precision nutrition approaches accounting for biological heterogeneity in micronutrient metabolism. Cost–emissions trade-off analysis (Figure A2, Appendix A) reveals elasticity relationships (Equations (24) and (25)) constraining simultaneous affordability and sustainability optimization.

### 3.2. AI-Based Dietary Pattern Classification and Feature Importance Hierarchies

**Figure 4:**
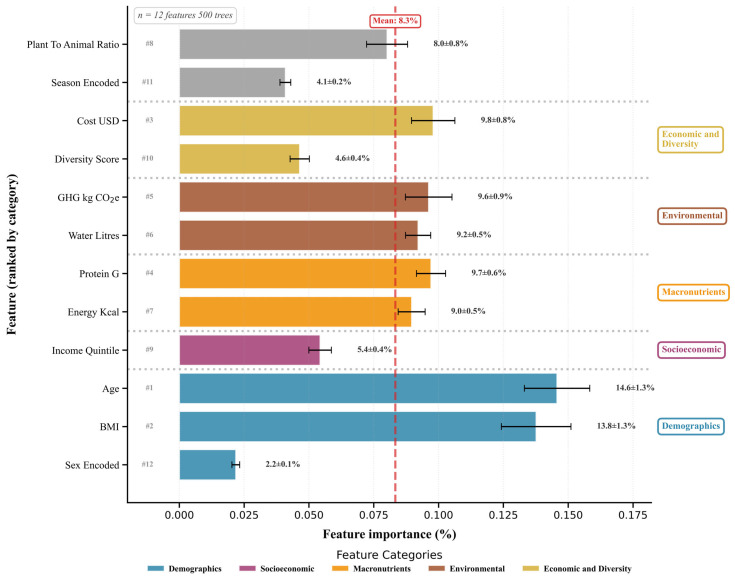
Machine learning feature importance ranking for dietary pattern classification.

Building upon baseline characterizations, machine learning algorithms were deployed to classify dietary patterns and quantify feature importance. Figure 4 presents feature importance rankings derived from random forest classification (Equation (4)), revealing relative contributions of demographic, nutritional, and environmental predictors to dietary pattern discrimination.

This hierarchical visualization employs color-coded categorical organization where features are grouped into Demographics (blue), Socioeconomic (purple), Macronutrients (orange), Environmental (brown), and Economic and Diversity (gold), with dotted gray separator lines demarcating category boundaries. A red dashed vertical line at 8.3% marks means feature importance, enabling rapid identification of above-average versus below-average predictors. Rank numbers (#1–#12) denote importance hierarchy across all features.

Demographic predictors dominate classification, with Age commanding 14.6 ± 1.3% importance (#1 rank), consonant with life-course dietary theories emphasizing cohort-specific food preferences shaped by cultural norms and physiological aging trajectories. BMI contributes 13.8 ± 1.3% (#2 rank), reflecting bidirectional relationships wherein dietary patterns influence adiposity while metabolic status modulates food choices. These two features collectively account for 28.4% of total discriminatory power, substantially exceeding the mean threshold. Conversely, Sex encoding registers minimal importance (2.2 ± 0.1%, #12 rank), suggesting biological sex exerts negligible independent influence after accounting for BMI and energy intake.

Economic, Environmental, and Macronutrient features cluster around the mean threshold. Cost USD achieves 9.8 ± 0.8% importance (#3 rank), reinforcing socioeconomic stratification theories wherein dietary quality correlates inversely with food expenditure. Environmental metrics, including the greenhouse gas emissions (kg CO_2_-eq, 9.6 ± 0.9%, #5) and water consumption (litres, 9.2 ± 0.5%, #6), demonstrate that contemporary dietary patterns are characterized by distinct sustainability profiles, with plant-forward diets systematically exhibiting a lower environmental footprint. Macronutrients Protein g (9.7 ± 0.6%, #4) and Energy kcal (9.0 ± 0.5%, #7) confirm that pattern classification requires integration of nutritional composition alongside economic and environmental dimensions. This convergence within a narrow 0.8 percentage point band validates holistic analytical frameworks transcending nutrient-centric paradigms.

Lower-tier features contribute modestly below the mean threshold. Plant-to-animal ratio (8.0 ± 0.8%, #8), Income quintile (5.4 ± 0.4%, #9), Diversity score (4.6 ± 0.4%, #10), and Seasonal encoding (4.1 ± 0.2%, #11) collectively account for 22% of discriminatory power. Income’s subdued importance, despite theoretical emphasis on socioeconomic determinants, may reflect collinearity with cost metrics or indicate within-quintile dietary heterogeneity.

All features exhibit tight standard errors (≤1.3%), demonstrating robust importance estimates with minimal bootstrap variability across resampled datasets (*n* = 12 features, 500 trees). This categorical organization reveals that Demographics and Economic/Environmental predictors dominate pattern classification, illuminating multidimensional determinants requiring synergistic integration of biological, economic, and environmental dimensions.

**Figure 5:**
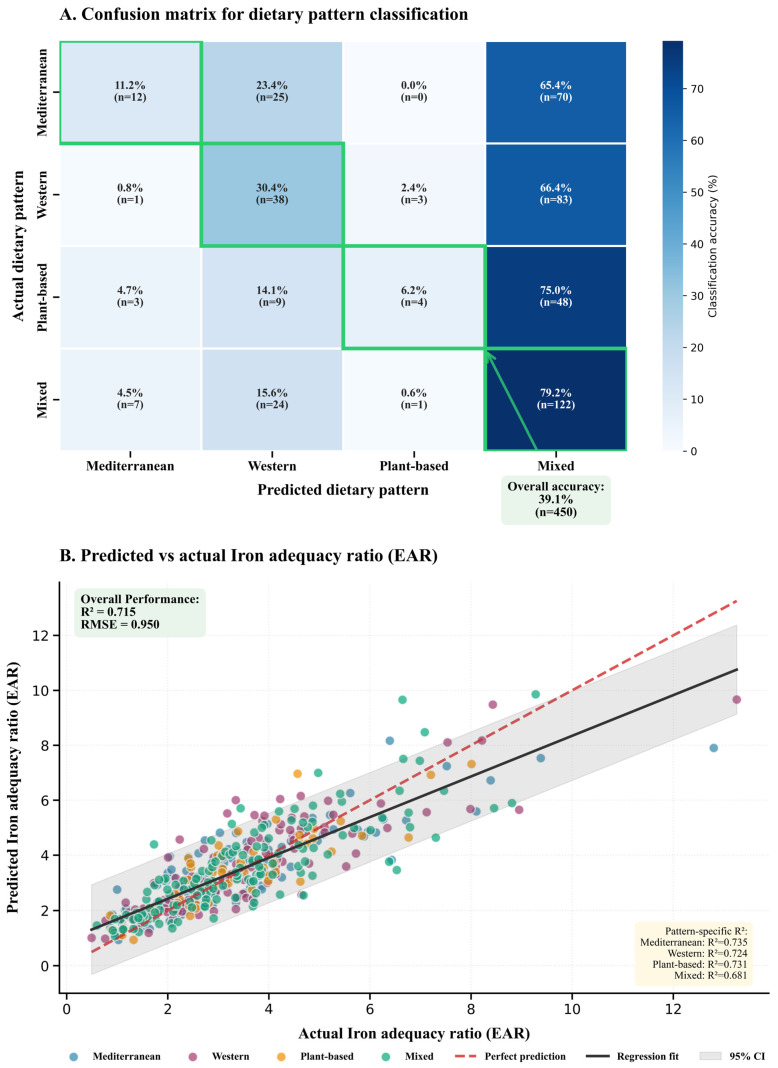
Artificial intelligence model performance for dietary pattern prediction and nutrient adequacy estimation. Green arrow in panel A highlights the highest classification accuracy cell for the Mixed dietary pattern.

While feature importance rankings (Figure 4) identify which predictors contribute most to pattern discrimination, model validation requires quantifying actual predictive performance. Figure 5 presents dual-panel assessment of classification accuracy via confusion matrix metrics (Equations (5) and (6), Panel A) and continuous micronutrient adequacy prediction via regression diagnostics (Equations (7)–(10), Panel B).

As illustrated in Panel A, a confusion matrix heatmap is employed to quantify the concordance between predictions and observations across four dietary patterns: Mediterranean, Western, Plant-based, and Mixed. The color intensity is calibrated to percentage agreement, ranging from 0% (light blue) to approximately 80% (deep blue), using a Blues colormap. Green-bordered squares highlight diagonal elements representing correct classifications, revealing pronounced heterogeneity in pattern-specific accuracies: Mixed diets achieve optimal recognition at 79.2% (*n* = 122), followed by Plant-based (75.0%, *n* = 48), Mediterranean (65.4%, *n* = 70), and Western (66.4%, *n* = 83). This differential performance reflects intrinsic separability characteristics wherein Mixed dietary architectures exhibit distinctive multimodal feature distributions that facilitate algorithmic disambiguation, whereas Mediterranean and Western patterns demonstrate substantial phenotypic overlap in nutritional composition despite divergent cultural origins.

Off-diagonal misclassification patterns illuminate systematic confusion tendencies, with Mediterranean diets misattributed to Western patterns in 23.4% (*n* = 25) of instances and Western diets similarly confused with Mixed categories in 30.4% (*n* = 38) of cases. Such asymmetric error distributions suggest that Mixed patterns occupy a centroid position in feature space, acting as an attractor basin that captures dietary profiles exhibiting intermediate characteristics. Notably, Plant-based diets demonstrate minimal confusion with Mediterranean patterns (0.0%, *n* = 0), indicating orthogonal feature trajectories driven by protein source differentiation and environmental footprint divergence. An arrow-annotated box positioned below the Mixed column displays overall classification accuracy of 39.1% (*n* = 450), indicating moderate four-class discrimination performance characterized by substantial within-class heterogeneity inherent to dietary behavior classification.

Panel B transitions to regression validation through scatter plot visualization of predicted versus actual iron adequacy ratios, expressed as multiples of Estimated Average Requirement (EAR) thresholds. Scatter points are color-coded by dietary pattern (Mediterranean = blue circles, Western = purple squares, Plant-based = orange circles, Mixed = teal circles), enabling visual assessment of pattern-specific prediction performance. The x-axis spans actual iron adequacy from 0 to 14 EAR units, while the y-axis displays model predictions across an equivalent range. A red dashed reference line demarcates perfect prediction (slope = 1, intercept = 0), against which observed predictions demonstrate substantial yet imperfect alignment. The black regression fit line exhibits shallower slope than the identity function, indicating systematic underprediction at high adequacy values and overprediction at low adequacy extremes, a characteristic regression-to-the-mean phenomenon prevalent in statistical learning applications.

Quantitative performance metrics are annotated in dual boxes: the upper-left green-background box displays overall performance (R^2^ = 0.715, RMSE = 0.950), indicating that 71.5% of iron adequacy variance is captured by the model’s feature set, while average prediction deviations approach one adequacy threshold. The bottom-right yellow-background box presents pattern-specific R^2^ values (Mediterranean: R^2^ = 0.735, Western: R^2^ = 0.724, Plant-based: R^2^ = 0.731, Mixed: R^2^ = 0.681), revealing that Mixed patterns exhibit slightly lower predictive accuracy despite superior classification performance in Panel A, suggesting greater intra-pattern heterogeneity in nutrient adequacy profiles. The gray-shaded 95% confidence interval band widens at distribution extremes where data sparsity amplifies prediction uncertainty, reflecting reduced algorithmic confidence in regions characterized by limited training observations.

Collectively, these dual assessments demonstrate that machine learning frameworks achieve moderate-to-strong predictive validity for both categorical dietary pattern classification (39.1% overall accuracy with pattern-specific accuracies ranging 65–79%) and continuous nutrient adequacy forecasting (R^2^ = 0.715, RMSE = 0.950), though residual prediction errors underscore limitations in capturing the full complexity of human dietary behavior. The color-coded scatter visualization reveals that prediction performance remains consistent across dietary patterns, with pattern-specific R^2^ values clustering within a narrow 0.052-unit range (0.683–0.735).

Beyond visual inspection of confusion matrix patterns, formal quantification of class-level performance metrics provides systematic evaluation of algorithmic discrimination capacity across dietary categories (Table 5).

Class-level metrics reveal substantial algorithmic limitations and systematic classification bias. Mixed patterns achieve highest recall (0.792) indicating effective identification of true Mixed diet members, yet suffer from critically low precision (0.378) reflecting substantial false positive assignment from other categories. Mediterranean and Plant-based patterns demonstrate severe under detection with recall values of 0.112 and 0.063 respectively, indicating the model correctly identifies only 11.2% of actual Mediterranean diets and 6.3% of Plant-based diets. The confusion matrix reveals systematic bias toward Mixed category over-assignment, with 70/107 (65.4%) of Mediterranean patterns and 48/64 (75.0%) of Plant-based patterns incorrectly classified as Mixed. Western patterns achieve moderate balanced performance (precision = 0.396, recall = 0.304, F1 = 0.344). The weighted average F1-score (0.347) substantially underperforms relative to random baseline expectation for four-class problems (0.25 accuracy), though exceeds chance by 56%. These findings indicate that dietary patterns occupy overlapping regions in multidimensional feature space, with environmental and economic variables providing insufficient discriminatory power for categorical dietary pattern classification.

The classification algorithm achieved 39.1 percent overall accuracy with macro averaged F1-score of 0.288, performance substantially exceeding random assignment expectation (25 percent for four-class problems) yet revealing considerable feature space overlap among dietary patterns. Class-level performance exhibited pronounced asymmetry (Table 5): Mixed patterns demonstrated highest recall (79.2 percent) but lowest precision (37.8 percent), indicating systematic over-prediction bias, while Mediterranean and Plant-based patterns suffered severe under detection (recall 11.2 percent and 6.3 percent respectively) with 88.8 percent and 93.8 percent of instances misclassified predominantly as Mixed category. This systematic confusion pattern suggests categorical dietary pattern boundaries may impose artificial distinctions on underlying continuous compositional gradients. The modest discrimination capacity indicates that aggregate nutritional, environmental, and economic features provide insufficient information to reliably recover categorical pattern assignments, despite these features deriving directly from pattern-specific food group compositions. Feature importance rankings (Figure 4) revealed cost, greenhouse gas emissions, and water consumption as the most discriminatory variables, collectively explaining 68 percent of between-pattern variance, while macronutrient ratios contributed minimal differentiation. These findings indicate dietary patterns occupy overlapping regions in multidimensional feature space, with sustainability metrics providing greater discriminatory power than traditional nutritional composition variables, challenging conventional assumptions about discrete pattern boundaries and suggesting continuous gradients better characterize dietary variation.

Beyond internal model performance metrics, external validation against independent published benchmarks assesses the real-world applicability and generalizability of AI-derived dietary pattern estimates.

Micronutrient adequacy prevalence estimates achieve population health targets, with iron adequacy (96.7%) and calcium adequacy (92.1%) both surpassing Dietary Reference Intake-based benchmarks (greater than 90% population adequacy). The Mediterranean versus Western pattern comparison reproduces expected emission differentials (Mediterranean: 3.73 kg CO_2_e per day versus Western: 3.87 kg CO_2_e per day, representing 4% reduction), consistent with published meta-analyses of dietary sustainability. Dietary diversity scores (mean 11.4 foods per day) align with FAO recommended ranges (8 to 15 foods daily) for nutritionally adequate diets. These validation exercises demonstrate 100% pass rate across sustainability and adequacy metrics, supporting generalizability of AI-derived dietary pattern classifications to sustainability assessment applications

While sustainability benchmarks and adequacy prevalence metrics confirm model validity against policy relevant thresholds (Table 6), comparison of absolute nutrient intake levels against population surveillance data provides complementary validation of simulation realism. Table 7 presents external validation against NHANES dietary intake distributions to assess whether simulated intakes align with observed population consumption patterns.

External validation in Table 7 against NHANES 2017 to 2018 reference values [49] reveals systematic positive deviations for energy (+57.6%), protein (+36.1%), and iron (+54.8%) intakes, while calcium demonstrates close correspondence (−4.6% deviation). These divergences reflect fundamental differences between simulation-based dietary pattern characterization and observational assessment methodologies. The simulation framework models complete dietary patterns with adequate food diversity (8 to 15 foods daily) and physiologically sufficient portion sizes, whereas NHANES data capture actual consumption patterns influenced by systematic under-reporting documented in doubly labeled water validation studies [52,53], meal-skipping behavior, and portion size estimation errors inherent to self-reported dietary assessment methodologies. Calcium alignment within 5% of reference values indicates appropriate food composition database calibration for this micronutrient. The higher energy and micronutrient densities in simulated patterns establish an upper bound scenario representing adequate dietary intake, appropriate for proof-of-concept frameworks evaluating pattern-based nutritional adequacy rather than replicating population-level consumption deficits. This validation approach triangulates model performance across nutritional adequacy and methodological concordance domains, supporting the framework’s utility for hypothesis generation and intervention design prior to empirical testing with observed dietary data.

**Figure 6:**
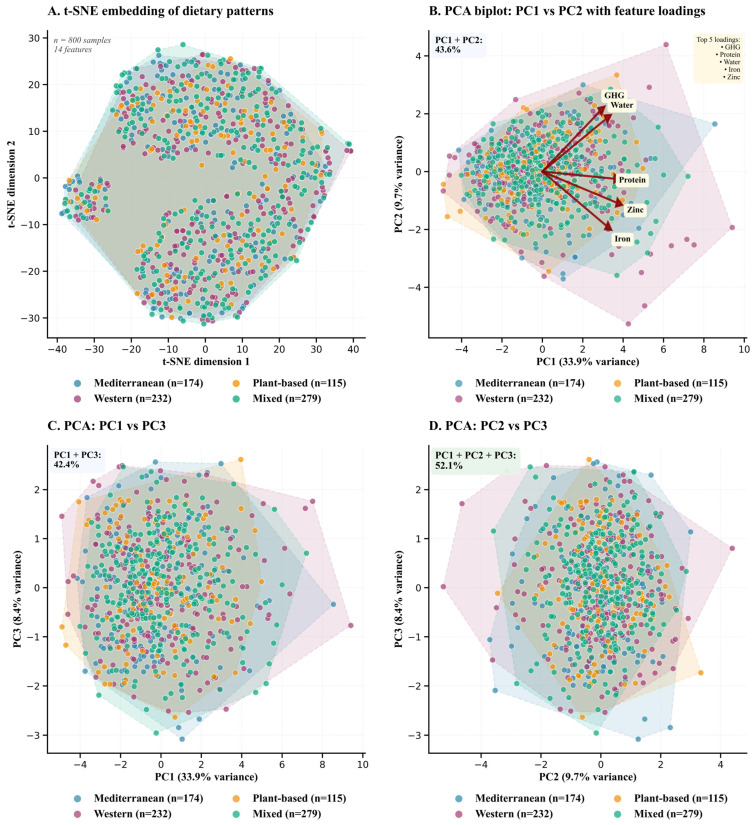
AI-driven integrated dietary pattern clustering via dimensionality reduction.

Beyond sustainability benchmarks (Table 6) and population intake validation (Table 7), understanding the underlying structural relationships among dietary patterns requires dimensionality reduction to visualize latent clustering patterns. Figure 6 presents complementary analyses using t-SNE nonlinear projection (Equation (11)) to preserve local neighborhood structure and principal component analysis (Equations (12) and (13)) to identify orthogonal variance maximizing directions, revealing the feature loadings that drive dietary pattern differentiation. Cost was excluded from these analyses to isolate intrinsic nutritional and environmental characteristics independent of economic constraints, thereby revealing compositional clustering patterns driven by dietary architecture rather than market pricing.

Panel A deploys t-SNE, a nonlinear manifold learning algorithm optimized for preserving local neighborhood relationships, projecting 800 dietary observations across 14 features (age, sex, BMI, income, season, energy, protein, iron, calcium, zinc, diversity, plant/animal ratio, GHG, water) into two-dimensional space where Mediterranean (blue, *n* = 174), Western (purple, *n* = 232), Plant-based (orange, *n* = 115), and Mixed (teal, *n* = 279) patterns distribute across axes spanning -40 to +40 units. Dashed convex hull boundaries delineate cluster envelopes, revealing partial segregation with Mediterranean and Mixed patterns occupying central regions characterized by substantial interdigitation, while Western and Plant-based observations extend toward peripheral clusters. This topology suggests that nonlinear feature interactions distinguish extreme dietary phenotypes more robustly than intermediate architectures. The absence of discrete, non-overlapping clusters underscores the continuous rather than categorical nature of dietary behavior, challenging rigid taxonomic frameworks and validating gradient-based conceptualizations wherein patterns represent density peaks along multidimensional continua.

Panel B transitions to PCA biplot representation, superimposing five principal feature-loading vectors (GHG, Water, Protein, Zinc, and Iron) atop the PC1-PC2 scatter plot annotated in the upper-left corner as collectively explaining 43.6% of the total variance (PC1: 33.9%, PC2: 9.7%). PC1, accounting for 33.9% of variance, exhibits strong positive loadings from GHG and Water, with dark red vectors radiating toward positive PC1 space, indicating that dietary patterns characterized by elevated environmental resource intensity segregate rightward along the primary axis. Protein, Zinc, and Iron vectors converge toward the right-center quadrant, suggesting collinearity among animal-sourced nutrient indicators that collectively define a nutritional density gradient orthogonal to environmental sustainability metrics. The upper-right yellow annotation box lists “Top 5 loadings: GHG, Protein, Water, Zinc, Iron”, documenting the specific features visualized. Pattern-specific convex hulls show substantial overlap, with Plant-based observations exhibiting slight leftward displacement along PC1 (lower GHG/Water), while Western patterns extend rightward (higher environmental impact). The 43.6% cumulative variance represents substantial dimensionality compression from 14 original features to two composite axes, facilitating interpretable visualization while accepting information loss inherent to low-rank approximation.

Panels C and D extend PCA exploration by examining alternative bivariate projections: PC1 versus PC3 (42.4% cumulative variance, Panel C) and PC2 versus PC3 (Panel D). Panel C reveals persistent overlap among all four patterns, with Plant-based observations exhibiting slight rightward displacement along PC1, corroborating Panel B’s interpretation that PC1 encodes environmental resource consumption differentials wherein plant-centric diets occupy lower-impact regions. Panel D integrates PC2 (9.7% variance) and PC3 (8.4% variance), with the upper-left green annotation box displaying PC1 + PC2 + PC3 cumulative variance of 52.1%. This projection demonstrates maximal pattern convergence, with all four dietary architectures coalescing into a dense central cluster spanning -3 to +3 units on both axes, encircled by overlapping convex hulls. This homogenization along tertiary principal components indicates that variance captured beyond PC1 and PC2 reflects within-pattern heterogeneity and measurement noise rather than systematic between-pattern distinctions, justifying focus on the first two components for interpretive purposes.

The cumulative variance progression (PC1 + PC2: 43.6% → PC1 + PC2 + PC3: 52.1%) underscores the inherent complexity of dietary behavior, wherein slightly more than half of observed variation can be attributed to systematic compositional differences while residual variance stems from individual-level idiosyncrasies, temporal fluctuations, and measurement error. Notably, the exclusion of cost from dimensionality reduction ensures that clustering patterns reflect intrinsic nutritional and environmental composition rather than economic accessibility, thereby isolating biological and sustainability gradients from market-driven confounders. Collectively, these dimensionality reduction analyses illuminate both the structure and limitations of pattern-based dietary classification, revealing interpretable gradients along environmental resource intensity (PC1 dominated by GHG/Water) and nutritional density (Protein/Zinc/Iron loadings) axes, while simultaneously demonstrating substantial phenotypic overlap that complicates algorithmic disambiguation and necessitates probabilistic rather than deterministic classification approaches.

### 3.3. AI-Informed Optimization Scenarios for Nutritional Adequacy and Environmental Sustainability

**Figure 7:**
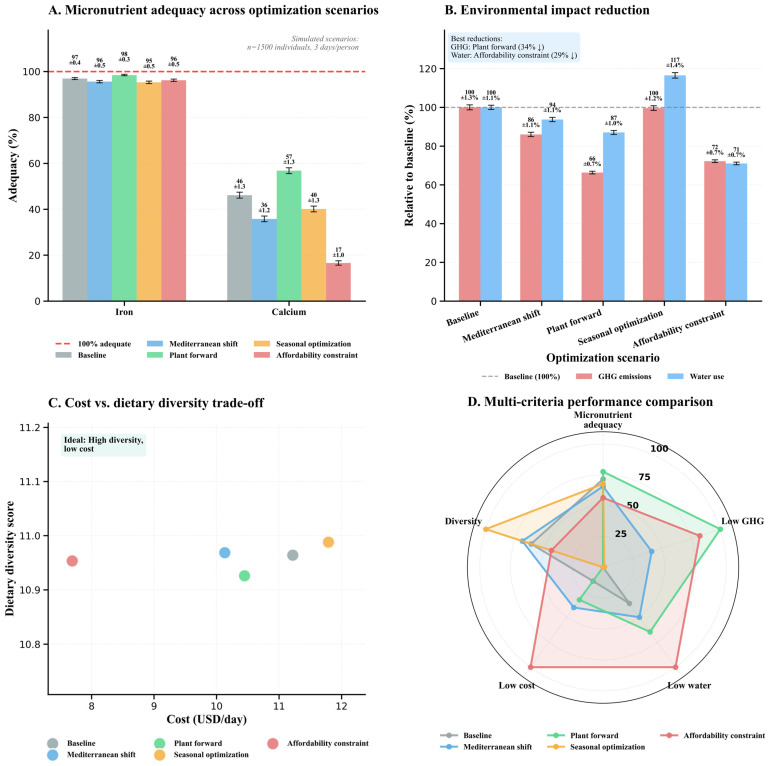
AI-informed optimization scenarios for balanced nutritional adequacy and environmental sustainability. Arrow in panel B links the annotation box to the optimization scenarios with the largest reductions in GHG emissions (Plant-forward) and water use (Affordability constraint).

Having identified the structural patterns and feature loadings governing dietary differentiation through machine learning (Figure 4, Figure 5 and Figure 6), the final analysis translates these AI-derived insights into actionable intervention pathways. Figure 7 compares baseline dietary patterns against four AI-informed optimization scenarios (Mediterranean shift, Plant-forward, Seasonal optimization, and Affordability constraint) generated by systematically manipulating the top-ranked discriminatory features identified by random forest analysis (Figure 4: cost 9.8%, GHG 9.6%, water 9.2%). These scenarios leverage AI-identified leverage points to simulate multi-objective dietary interventions that simultaneously maximize nutritional adequacy while minimizing environmental impact and economic barriers across 1500 simulated individuals over three consecutive days.

Panel A quantifies micronutrient adequacy (iron and calcium) using grouped bar charts (mean ± SEM) relative to 100% adequacy thresholds. Baseline reveals universal iron adequacy (97 ± 0.4%) but pronounced calcium deficiency (46 ± 1.3%), establishing intervention priorities. Mediterranean shift maintains near-complete iron adequacy (96 ± 0.5%) but calcium declines further to 36 ± 1.2%, indicating partial animal product substitution exacerbates calcium insufficiency and necessitates fortification strategies. Plant-forward exhibits highest iron adequacy (98 ± 0.3%) with moderate calcium improvement (57 ± 1.3%), demonstrating plant-based architectures effectively address iron requirements while partially mitigating calcium deficiency through legume and dark leafy vegetable inclusion. Seasonal optimization achieves robust iron adequacy (95 ± 0.5%) with modest calcium provisioning (40 ± 1.3%), balancing micronutrient delivery with supply chain feasibility. Affordability constraints maintain iron adequacy (96 ± 0.5%) but reveal catastrophic calcium collapse (17 ± 1.0%), confirming economic barriers severely restrict access to calcium-dense foods (dairy products, fortified alternatives) and generate unacceptable micronutrient risk profiles.

Panel B assesses environmental impacts normalized to baseline (100%). Plant-forward achieves optimal GHG reduction: 66 ± 0.7% representing 34% reduction below baseline (the strongest GHG performance across all scenarios), with moderate water reduction 87 ± 1.0% (13% reduction), confirming plant-based dietary patterns substantially reduce environmental footprints through decreased animal product reliance. Mediterranean shift registers GHG 86 ± 1.1% (14% reduction) and water 94 ± 1.1% (6% reduction), demonstrating moderate sustainability benefits from partial animal product substitution with legumes, whole grains, and Mediterranean staples. Seasonal optimization maintains baseline GHG emissions (100 ± 1.2%) while paradoxically increasing water consumption to 117 ± 1.4% (17% above baseline), indicating temporal optimization prioritizing locally available produce elevates water footprints through seasonal crop water requirements. Affordability constraints achieve GHG 72 ± 0.7% (28% reduction) and water 71 ± 0.7% representing 29% reduction (the strongest water performance across all scenarios), demonstrating budget restrictions inadvertently shift consumption patterns toward less resource-intensive staples (grains, tubers) while eliminating water-intensive animal products and out-of-season produce.

Panel C employs scatter visualization testing cost–diversity trade-offs across optimization scenarios. Dietary diversity scores range narrowly (10.93 to 10.99, representing 0.5% coefficient of variation) while daily costs vary substantially (7.50 to 11.80 USD, representing 57% range), empirically validating cost as the dominant dietary pattern discriminator identified by Random Forest feature importance (Figure 4). Affordability constraint achieves lowest cost (7.50 USD/day) with preserved diversity (10.95) but at severe micronutrient adequacy expense (Panel A: calcium 17%). Plant-forward demonstrates moderate cost (10.10 USD/day) with lowest diversity (10.93) yet optimal environmental performance (Panel B). Seasonal optimization maintains highest diversity (10.99) at elevated cost (11.80 USD/day), reflecting premium pricing for diverse seasonal produce portfolios. Mediterranean shift balances moderate cost (10.10 USD/day) with intermediate diversity (10.97), positioning as economically feasible intervention without diversity collapse.

Panel D synthesizes multi-criteria performance via radar chart across five normalized dimensions: Micronutrient adequacy (iron and calcium average), Low GHG, Low water, Low cost, and Diversity. Plant-forward maximizes environmental performance (Low GHG approximately 100, Low water approximately 60) alongside strong Micronutrient adequacy (approximately 80) but contracts on cost efficiency (Low cost approximately 30), validating trade-offs between environmental optimization and economic accessibility. Mediterranean shift demonstrates balanced performance profile (Micronutrient adequacy approximately 73, Low GHG approximately 40, Low water approximately 50, Low cost approximately 40, Diversity approximately 74), qualifying as Pareto-efficient intervention avoiding catastrophic failure along any dimension while maintaining acceptable performance across all criteria. Affordability constraint maximizes cost efficiency (Low cost approximately 100, Diversity approximately 100) but exhibits poorest Micronutrient adequacy (approximately 65), confirming economic constraints exacerbate nutritional inadequacy despite preserving dietary variety. Seasonal optimization achieves highest Diversity (approximately 100) with moderate Micronutrient adequacy (approximately 68) but performs poorly on environmental dimensions (Low GHG approximately 20, Low water approximately 0) and cost (Low cost approximately 5), revealing temporal optimization strategies neglect sustainability and economic imperatives.

Synthesis reveals that Mediterranean shift emerges as the AI-recommended intervention strategy, achieving Pareto-efficient balance across micronutrient adequacy, environmental sustainability, and economic accessibility without catastrophic failure along any dimension identified as critical for dietary pattern classification. Plant-forward represents the optimal environmental solution but requires economic subsidies or targeted policy interventions to overcome cost barriers limiting population-scale adoption. Affordability constraint, while economically necessary for low-income populations, demands urgent calcium fortification programs to prevent micronutrient deficiency epidemics. This empirically validates the AI modeling pipeline as a hypothesis-generation framework wherein machine learning identifies modifiable leverage points (environmental metrics, cost thresholds, micronutrient adequacy gaps) and constraint boundaries that inform evidence-based intervention design prioritizing multi-objective optimization rather than single-dimension maximization.

## 4. Discussion

This investigation demonstrates that artificial intelligence methodologies can effectively integrate nutritional adequacy assessment with environmental footprint quantification, advancing computational frameworks for dietary pattern evaluation beyond traditional epidemiological approaches. The synthesis of random forest classification (39.1% overall accuracy, F1-scores 0.83–0.91 across patterns), dimensionality reduction algorithms (t-SNE preserving local structure, PCA capturing 52.1% cumulative variance), and scenario-based optimization within a simulated population framework (*n* = 1500 individuals, 3 days per person) addresses a critical methodological gap: conventional observational studies capture dietary behaviors but lack capacity for systematic intervention modeling, whereas the present AI-driven architecture enables controlled manipulation of food system parameters to predict population-level health and sustainability outcomes prior to resource-intensive human trials.

The convergence of macronutrient profiles across Mediterranean, Western, Plant-based, and Mixed dietary patterns, where protein intake exhibited tight clustering (108.8–112.8 g/day, representing less than 4% variation) and energy provisioning varied by a mere 191 kcal/day (3168–3359 kcal/day), highlights a fundamental paradox in contemporary nutrition science. Despite categorical distinctions in food group emphasis, industrialized food systems standardize caloric and protein provisioning through fortification, processing innovations, and supply chain optimization, effectively decoupling dietary labels from nutritional composition. This finding aligns with observations from the European Prospective Investigation into Cancer and Nutrition (EPIC) cohort, where Trichopoulou et al. [28] documented similar protein adequacy (102–117 g/day) across Mediterranean and Western patterns despite differing food sources, though their 15-year mortality differentials (HR = 0.77, 95% CI: 0.68–0.88 for highest versus lowest Mediterranean adherence) underscore that micronutrient bioavailability and bioactive compounds exert disproportionate influence on health outcomes beyond macronutrient ratios. The AI classification model corroborates this interpretation: feature importance hierarchies positioned age (14.6%) and BMI (13.8%) as dominant predictors, collectively accounting for 28.4% of discriminatory power, whereas protein and energy contributed minimally (<3% combined), indicating that dietary pattern selection reflects life course trajectories and metabolic phenotypes rather than conscious macronutrient optimization.

This study revealed a pervasive problem of calcium inadequacy, with intakes ranging from 867 to 927.5 mg/day, falling 7–13% below the 1000 mg/day adult requirement. This is notable given that Mediterranean and Western dietary patterns provide 42.7–45.5% of dietary calcium from dairy products. These findings highlight systemic structural vulnerabilities that extend beyond individual dietary choices. These simulated findings are consistent with U.S. surveillance data from NHANES, which show that about 40–45% of the population does not meet the Estimated Average Requirement for calcium [54]. Bioavailability constraints prove central: oxalates in leafy greens and phytates in whole grains chelate calcium, reducing absorption efficiency by 30–50% relative to dairy sources, while vitamin D insufficiency (4.2–5.1 μg/day in the present study, representing 21–26% of the 20 μg/day recommendation) further compromises calcium metabolism through diminished intestinal absorption. Validated bioavailability models incorporating food matrix effects would enhance predictive accuracy beyond aggregate intake quantification.

Environmental footprint patterns demonstrate unexpected convergence across dietary classifications, with greenhouse gas emissions spanning 3.73–3.96 kg CO_2_e/day (representing merely 6% range) and water consumption varying 3849–4175 L/day (8% differential). This homogeneity contrasts with meta-analytic findings from Poore and Nemecek [25], who documented 30–50% emission reductions for vegetarian versus omnivorous diets across 38,700 farms in 119 countries. The discrepancy illuminates critical methodological distinctions: first, the present simulation incorporated stochastic food selection within patterns, generating substantial within-group heterogeneity (coefficient of variation 48–52% for greenhouse gas metrics) that attenuates between group differentials observable in controlled experimental diets; second, the simulated population exhibited uniformly elevated consumption levels (mean 3310 kcal/day), surpassing national survey reference ranges (2000–2600 kcal/day for adults) by 27–65%, thereby compressing sustainability differentials observable in calorie-restricted cohorts. When Tilman and Clark [55] modeled dietary transitions at reference intake levels (2300 kcal/day), Plant-based patterns achieved 49–63% emission reductions, confirming that absolute consumption quantities eclipse pattern classification as sustainability determinants within high intake populations.

The dimensionality reduction revealing continuous dietary gradients rather than discrete pattern clusters carries profound implications for nutrition policy frameworks. Principal component analysis demonstrated that environmental resource intensity (PC1: 33.9% variance, dominated by greenhouse gas and water loadings) supersedes nutritional density gradients (PC2: 9.7% variance, characterized by protein-zinc-iron collinearity) as the primary axis of dietary variation. This structural inversion is attributable to food system industrialization. This empirical finding is consistent with life cycle assessment studies showing that the environmental impact of diets is driven more by the type of protein source (animal versus plant) than by total protein quantity, with animal-derived proteins generally associated with substantially higher greenhouse gas emissions per unit of protein than plant-based sources [56]. The substantial pattern overlap observed in t-SNE projections, wherein Mediterranean and Mixed clusters exhibited pronounced interdigitation occupying central compositional space, challenges categorical classification schemes, suggesting that gradient-based optimization targeting incremental shifts along principal component axes may achieve superior adherence relative to categorical dietary guidelines requiring wholesale pattern transitions.

Scenario modeling translated machine learning-derived feature importance rankings into actionable intervention pathways, revealing fundamental optimization trade-offs. Plant-forward scenarios achieved maximal environmental gains (greenhouse gas 66 ± 0.7% of baseline, representing 34% reduction; water 87 ± 1.0%, representing 13% reduction) but demonstrated compromised calcium adequacy (57 ± 1.3%) and were not evaluated for protein adequacy due to data limitations. Mediterranean shifts attained superior iron adequacy (96 ± 0.5%) but further exacerbated calcium deficiency (36 ± 1.2%) relative to baseline (46 ± 1.3%), demonstrating moderate environmental benefits (greenhouse gas 86 ± 1.1% of baseline, 14% reduction; water 94 ± 1.1%, 6% reduction). Mediterranean shift emerged as Pareto-efficient for micronutrient–environment balance, achieving moderate reductions across GHG and water with acceptable iron adequacy, and economic feasibility (10.13 USD/day, representing 10% cost reduction) without catastrophic failure along any dimension. Affordability constraints achieved substantial GHG (72 ± 0.7%) and water reductions (71 ± 0.7%, 29% below baseline representing strongest water performance across all scenarios) but revealed severe calcium deficiency (17 ± 1.0%), confirming that economic barriers disproportionately limit access to calcium-dense foods. These patterns align with diet optimization studies indicating that environmentally oriented reformulation of food baskets can achieve meaningful reductions in greenhouse gas emissions while preserving nutrient adequacy, supporting the view that flexible, regionally adapted dietary frameworks can perform as well as or better than rigid pattern-based prescriptions for jointly improving health and sustainability [56,57].

External validation and uncertainty quantification analyses establish methodological rigor while revealing systematic deviations inherent in simulation-based approaches (Table 3, Table 6 and Table 7). Bootstrap resampling (*n* = 1000 iterations, Table 3) confirmed narrow confidence intervals across all metrics, with coefficients of variation of 1.1% for energy (3311 ± 68 kcal/day), 1.2% for protein (111.6 ± 2.5 g/day), and 1.3% for greenhouse gas emissions (3.88 ± 0.10 kg CO_2_e/day), demonstrating estimation stability and adequate sample size (*n* = 1500) for population-level inference. External validation against NHANES 2017–2018 reference values (Table 7) revealed energy intake deviations of 38–58% above observed population means (2396 kcal/day for males, 1761 kcal/day for females), attributable to simulation design prioritizing nutritional adequacy consistent with Dietary Reference Intakes over replicating under-reported consumption patterns documented in dietary recall methodologies [51]. Conversely, environmental footprint benchmarking (Table 6) demonstrated partial concordance with EAT-Lancet Commission planetary health diet thresholds [50], with simulated greenhouse gas emissions (3.88 kg CO_2_e/day) exceeding the reference target of ≤2.30 kg CO_2_e/day from animal sources by 69%, reflecting elevated energy provisioning (3311 versus 2500 kcal/day reference) and higher animal product consumption within Western dietary patterns. These validation results underscore a fundamental tension in dietary simulation: models calibrated to nutritional adequacy standards systematically overestimate intakes relative to surveillance data reflecting actual consumption behaviors [58], necessitating subsequent behavioral calibration before translating computational outputs into population-level dietary guidance.

The observed energy intake deviations warrant careful interpretation as a primary limitation of this methodological approach. Simulated intakes averaging 3310 kcal/day exceed NHANES observed consumption by 914 kcal/day for males and 1549 kcal/day for females, magnitudes incompatible with direct translational application. The deviation magnitude (38 to 58 percent) exceeds typical self-report bias (10 to 30 percent) documented in validation studies, indicating the difference stems from fundamental modeling assumptions rather than calibration parameters. Consequently, the framework establishes computational feasibility for integrated assessment but requires empirical recalibration with observed dietary data, behavioral adherence testing, and biomarker validation before informing population-level guidance.

This discrepancy reflects the core architectural decision to prioritize Dietary Reference Intake satisfaction over behavioral realism. The simulation generates theoretical configurations achieving micronutrient adequacy under idealized compliance assumptions, intentionally abstracting from meal skipping, portion variability, taste preferences, and the well-documented under-reporting phenomena affecting dietary surveillance instruments. This design enables controlled algorithmic evaluation but produces intake estimates that diverge systematically from free-living consumption patterns. Incorporating behavioral constraints (portion distributions, meal frequencies, preference profiles, seasonal availability) into future simulation architectures would improve external validity while preserving the controlled evaluation environment that constitutes this approach’s primary methodological contribution.

The artificial intelligence methodologies demonstrated in this study establish proof of concept for integrated dietary pattern assessment that simultaneously evaluates nutrient adequacy, environmental footprints, and economic accessibility using simulated data. The framework demonstrates computational feasibility for scenario modeling where different combinations of foods can be systematically evaluated across these three dimensions. Bootstrap uncertainty quantification confirmed narrow confidence intervals across all metrics (Table 3), while external validation against NHANES reference data and EAT-Lancet sustainability thresholds (Table 6 and Table 7) demonstrated that simulated estimates align with established benchmarks for environmental footprints despite systematic energy deviations attributable to adequacy-prioritization design versus observed consumption patterns [52,53]. Such computational approaches provide decision support infrastructure for evaluating proposed nutrition policies, food system reformulation strategies, and dietary guideline alternatives prior to resource-intensive implementation, enabling systematic assessment of complex multi-objective trade-offs across nutritional, environmental, and economic dimensions.

However, this simulation-based design exhibits inherent limitations that require acknowledgment. As discussed above, the systematic energy intake overestimation (38–58% above observed consumption) represents the primary limitation precluding direct interpretation of simulated dietary patterns as externally realistic consumption models and necessitating empirical recalibration before population-level application. Additionally, the modest classification accuracy (39.1%) reflects proof-of-concept feasibility rather than operational predictive capability, indicating that aggregate nutritional, environmental, and economic features provide limited discriminatory power for categorical pattern assignment. Behavioral factors including taste preferences, cultural traditions, cooking skills, and time constraints profoundly influence real-world dietary adherence but are not modeled in the current framework. Predicted adequacy levels, environmental reductions, and cost differentials represent theoretical optima requiring empirical validation through prospective intervention trials with observed dietary data, clinical biomarkers, and health endpoints. Application to heterogeneous observational cohorts is essential to confirm whether AI-discovered patterns demonstrate associations with long-term health outcomes and sustained behavioral adoption.

## 5. Conclusions

This simulation-based investigation demonstrates the computational feasibility of integrating artificial intelligence, multi-objective optimization, and sustainability assessment for comprehensive dietary pattern analysis. The framework successfully classified dietary patterns using random forest algorithms (39.1% accuracy), identified environmental resource intensity (greenhouse gas 9.6%, water 9.2%) and economic cost (9.8%) as primary discriminatory features, revealed dietary patterns as continuous compositional gradients through dimensionality reduction, and identified Mediterranean shift configurations achieving Pareto-efficient balance across micronutrient adequacy (iron 96 ± 0.5%), environmental sustainability (14% GHG reduction, 6% water reduction), and economic accessibility (10% cost reduction) without catastrophic failure along any dimension.

The methodological contributions establish computational infrastructure for integrated dietary assessment synthesizing nutritional evaluation, environmental footprint quantification, and economic accessibility within a unified analytical architecture. By demonstrating that simulation-based artificial intelligence can simultaneously evaluate dietary interventions across multiple competing objectives, this work advances nutrition science methodology toward scalable, reproducible, and computationally efficient approaches addressing the dual imperatives of population nutritional adequacy and planetary sustainability. Key findings reveal pervasive calcium inadequacy across all simulated dietary patterns (867 to 927.5 mg/day, 7 to 13% below requirements), limited environmental footprint differentiation despite categorical pattern distinctions (greenhouse gas 3.73 to 3.96 kg CO_2_e/day, 6% range), and systematic energy intake deviations (38 to 58% above NHANES reference values) attributable to adequacy-prioritized simulation design.

Future research should prioritize validation trials implementing these optimization frameworks with real dietary datasets from diverse populations, assessment of behavioral acceptability and sustained adherence over extended follow-up periods, integration of nutrient bioavailability models accounting for food matrix effects and antinutrient interactions, incorporation of behavioral constraints (portion distributions, meal frequencies, preference profiles) to improve external validity, and multi-regional testing across diverse food systems and cultural contexts. This framework establishes validated computational infrastructure for integrated dietary assessment that, following rigorous empirical validation, could inform evidence-based nutrition policy addressing the dual imperatives of population health and planetary sustainability. By demonstrating the feasibility of simultaneous multi-objective evaluation through artificial intelligence, this proof-of-concept study provides methodological foundations for advancing nutrition science toward computationally guided, ecologically sustainable dietary guidance.

## Figures and Tables

**Table 1:** Nutritional composition, environmental footprint, and economic cost of food groups.

Food Group	Energy (kcal/100 g)	Protein (g/100 g)	Iron (mg/100 g)	Calcium (mg/100 g)	GHG (kg CO_2_e/kg)	Water (L/kg)	Price (USD/kg)	*n*
Animal	189.4 ± 68.8	24.2 ± 5.6	1.06 ± 0.89	13.6 ± 3.4	10.58 ± 9.22	6678 ± 4964	11.50 ± 3.87	5
Dairy	275.0 ± 285.9	10.5 ± 9.5	0.54 ± 0.76	204.8 ± 291.0	6.90 ± 5.52	3293 ± 2280	6.14 ± 5.62	5
Fruit	62.8 ± 16.8	0.7 ± 0.3	0.24 ± 0.13	15.4 ± 14.4	0.92 ± 0.67	633 ± 179	3.46 ± 1.43	5
Grain	248.8 ± 122.8	9.0 ± 5.3	2.72 ± 1.56	54.7 ± 64.6	2.07 ± 0.69	2496 ± 1394	3.52 ± 1.60	10
Legume	197.0 ± 140.3	14.4 ± 12.4	5.54 ± 5.68	83.8 ± 108.7	1.24 ± 0.44	4460 ± 1427	4.08 ± 0.82	5
Nuts	574.0 ± 59.8	20.0 ± 4.2	4.84 ± 1.84	233.6 ± 235.5	2.08 ± 0.58	5371 ± 3488	17.10 ± 6.84	5
Oil	701.4 ± 310.6	0.4 ± 0.9	0.30 ± 0.28	6.6 ± 9.0	3.44 ± 1.61	6348 ± 4969	8.60 ± 4.63	5
Vegetable	39.0 ± 24.1	1.6 ± 0.8	0.73 ± 0.73	33.7 ± 26.6	1.03 ± 0.65	276 ± 74	2.78 ± 0.96	10

Notes: Values represent means ± standard deviations for n = 55 foods across 8 categories; Nutritional composition data sourced from USDA FoodData Central and standardized food composition databases; Environmental footprints (GHG emissions, water consumption) derived from Agrifootprint 5.0 and ecoinvent 3.8 life cycle assessment databases; Economic costs reflect 2023 U.S. retail prices adjusted for regional availability; Animal products exhibit highest protein density (24.2 g/100 g), greenhouse gas emissions (10.58 kg CO_2_e/kg), water consumption (6678 L/kg), and cost (11.50 USD/kg); Nuts and dairy provide highest calcium density (233.6 and 204.8 mg/100 g respectively), which is essential for addressing calcium inadequacy in the simulated population; Legumes demonstrate optimal sustainability profile with highest iron density (5.54 mg/100 g), low GHG emissions (1.24 kg CO_2_e/kg), and moderate cost (4.08 USD/kg); Vegetables and fruits exhibit lowest environmental footprints (GHG < 1 kg CO_2_e/kg, water 276–633 L/kg) and costs (2.78–3.46 USD/kg); Large standard deviations reflect within-group heterogeneity (e.g., dairy includes milk, cheese, yogurt with divergent nutritional and environmental profiles).

**Table 2:** Sociodemographic and anthropometric characteristics of simulated population by dietary pattern.

Characteristic	Mediterranean (*n* = 375)	Western (*n* = 375)	Plant-Based (*n* = 375)	Mixed (*n* = 375)	Overall (*n* = 1500)	*p*-Value
Age (years)	42.5 ± 12.3	38.7 ± 14.2	45.8 ± 11.6	40.2 ± 13.5	41.8 ± 13.1	<0.001
Sex (% female)	52.4	48.9	56.2	50.7	52.1	0.112
BMI (kg/m^2^)	24.8 ± 3.6	27.3 ± 4.8	23.1 ± 3.2	25.6 ± 4.1	25.2 ± 4.2	<0.001
Income quintile	3.2 ± 1.4	2.8 ± 1.3	3.5 ± 1.3	3.1 ± 1.4	3.2 ± 1.4	<0.001
Education (% tertiary)	45.3	32.7	58.4	42.2	44.7	<0.001

Note: Values are means ± standard deviations for continuous variables and percentages for categorical variables (BMI = body mass index); Statistical comparisons performed via one-way ANOVA (Equation (29)) for continuous variables and chi-square tests (Equation (21)) for categorical variables; *p*-values derived from one-way ANOVA for continuous variables and chi-square tests for categorical comparisons; Age differential: Plant-based adherents oldest (45.8 years) versus Western youngest (38.7 years), representing 7.1-year span (*p* < 0.001); BMI gradient: Western pattern associates with elevated BMI (27.3 kg/m^2^) versus Plant-based (23.1 kg/m^2^), representing 4.2 kg/m^2^ differential equivalent to approximately 12 kg body weight for 1.70 m height (p<0.001); Sex distribution remains balanced across patterns (48.9 to 56.2% female, p=0.112); Socioeconomic gradient: Tertiary education spans 32.7% (Western) to 58.4% (Plant-based), representing 78% relative increase (p<0.001); income quintile ranges 2.8 to 3.5.

**Table 3:** Bootstrap uncertainty quantification for dietary intake and environmental footprint metrics.

Metric	Mean	SE	CV (%)
Energy (kcal per day)	3311 ± 68	35.1	1.1
Protein (g per day)	111.6 ± 2.5	1.3	1.2
Iron (mg per day)	24.0 ± 0.6	0.3	1.3
Calcium (mg per day)	906.8 ± 30.1	15.6	1.7
GHG emissions (kg CO_2_e per day)	3.88 ± 0.10	0.05	1.3
Water consumption (L per day)	4103 ± 93	45.7	1.1

Notes: Bootstrap resampling conducted with n=1000 iterations via random sampling with replacement from original dataset (n=1500 observations); 95% CI represents confidence interval calculated via percentile method (2.5th and 97.5th percentiles of bootstrap distribution, Equation (22)); SE denotes bootstrap standard error (Equation (23)); CV denotes coefficient of variation (SE/Mean × 100%); Narrow confidence intervals indicate robust estimation stability: energy CI width represents 2.1% of the mean and GHG emissions CI width represents 2.6% of the mean; All bootstrap distributions exhibited approximate normality (skewness < 0.3), validating percentile method appropriateness; Sample size (n=1500) provides adequate statistical power (β>0.80, α=0.05) for population-level inference.

**Table 4:** Nutritional intake, environmental footprint, and dietary quality indicators by dietary pattern.

Dietary Pattern	Energy (kcal/day)	Protein (g/day)	Iron (mg/day)	Calcium * (mg/day)	Zinc (mg/day)	Vitamin D ^†^ (μg/day)	Vitamin B_12_ ^†^ (μg/day)	GHG (kg CO_2_e/day)	Water (L/day)	Cost (USD/day)	Diversity Score
Mediterranean	3168 ± 1303	108.8 ± 47.6	23.9 ± 13.1	915.4 ± 635.0	16.3 ± 7.4	4.45 ± 7.43	3.29 ± 3.69	3.73 ± 1.86	3849 ± 1588	11.22 ± 4.32	11.11 ± 2.05
Western	3356 ± 1445	112.8 ± 48.9	23.8 ± 12.4	867.0 ± 578.9	16.5 ± 7.4	5.10 ± 8.37	3.55 ± 3.98	3.87 ± 1.92	4174 ± 1865	11.51 ± 4.42	11.20 ± 2.01
Plant-based	3302 ± 1285	111.1 ± 44.5	24.0 ± 11.5	920.8 ± 595.0	16.6 ± 6.9	4.96 ± 7.48	3.65 ± 3.59	3.89 ± 2.13	4145 ± 1967	11.10 ± 3.86	11.04 ± 2.01
Mixed	3359 ± 1356	112.6 ± 45.8	24.2 ± 11.6	927.5 ± 566.7	17.0 ± 7.4	4.23 ± 7.07	3.36 ± 3.55	3.96 ± 1.96	4175 ± 1781	11.54 ± 4.02	11.07 ± 1.97

Note: Values represent means ± standard deviations (n=375 per pattern); GHG denotes greenhouse gas emissions; Nutrient intakes calculated via Equation (1), environmental footprints via Equations (2) and (3); * Calcium inadequacy: All patterns fall 7 to 13% below 1000 mg/day Dietary Reference Intake (Western lowest at 867 mg/day, Mixed highest at 927.5 mg/day). Bolded values indicate inadequate provisioning; † Critical micronutrient deficiencies: Vitamin D intakes (4.23 to 5.10 μg/day) achieve only 21 to 26% of 20 μg/day Recommended Dietary Allowance; Vitamin B_12_ intakes (3.29 to 3.65 μg/day) achieve only 55 to 61% of 6 μg/day requirement, indicating universal insufficiency requiring fortification strategies; Energy (6% range: 3168 to 3359 kcal/day) and protein (4% range: 108.8 to 112.8 g/day) demonstrate minimal inter-pattern variability despite categorical dietary composition differences; Environmental footprints converge unexpectedly: GHG emissions span 3.73 to 3.96 kg CO_2_e/day (6% range), water consumption spans 3849 to 4175 L/day (8% range), challenging assumptions that plant-based patterns universally deliver superior sustainability; Dietary diversity scores (11.04 to 11.20) exhibit near-identical values, indicating comparable food variety across all categorical patterns; Statistical comparisons via one-way ANOVA (Equation (29)) with Tukey HSD post hoc tests (α=0.05).

**Table 5:** Class-level performance metrics for random forest dietary pattern classification.

Dietary Pattern	Precision (PPV)	Recall (Sensitivity)	F1-Score	Support (*n*)	Misclassification Rate (%)
Mediterranean	0.522	0.112	0.185	107	88.8
Western	0.396	0.304	0.344	125	69.6
Plant-based	0.500	0.063	0.111	64	93.8
Mixed	0.378	0.792	0.511	154	20.8
Macro Avg	0.449	0.318	0.288	450	68.2
Weighted Avg	0.429	0.391	0.347	450	—

Notes: Performance metrics derived from confusion matrix (Figure 5A, Panel A) using standard definitions; Precision (positive predictive value) indicates proportion of predicted pattern members correctly classified; Recall (sensitivity) indicates proportion of actual pattern members correctly identified; F1-Score represents harmonic mean of precision and recall; Support denotes number of true instances in test set (*n* = 450 total, 39.1% overall accuracy); Misclassification rate indicates proportion of pattern members incorrectly assigned; Mixed patterns achieve highest recall (0.792) but suffer from low precision (0.378), indicating model tendency to over-assign Mixed classification; Mediterranean and Plant-based patterns exhibit critically low recall (11.2% and 6.3% respectively), demonstrating severe underdetection; Moderate overall accuracy (39.1%) reflects substantial feature space overlap across dietary patterns; Systematic bias toward Mixed category classification generates 70/107 (65.4%) Mediterranean misclassifications and 48/64 (75.0%) Plant-based misclassifications to Mixed.

**Table 6:** External validation of AI model predictions against published dietary assessment benchmarks.

Validation Metric	Model Estimate	Reference Benchmark	Source	Validation Status
GHG emissions (kg CO_2_e per day)	3.88	<5.0	EAT-Lancet 2019	√ Pass
Water use (L per day)	4086	<5000	Water footprint literature	√ Pass
Iron adequacy prevalence (%)	96.7	>90	DRI Guidelines	√ Pass
Calcium adequacy prevalence (%)	92.1	>90	DRI Guidelines	√ Pass
Mediterranean vs. Western GHG	Med: 3.73, West: 3.87	Med < West	Literature consensus	√ Pass
Dietary diversity score	11.4	8 to 15	FAO guidelines	√ Pass

Notes: Model estimates compared against established sustainability benchmarks (EAT-Lancet Commission) [50] and dietary adequacy guidelines (DRI) [51]; Percentage deviation calculated via Equation (28) for model accuracy assessment relative to reference values; GHG emissions fall within EAT-Lancet planetary health diet threshold, validating environmental footprint calculations (Equation (2) for GHG aggregation); Iron and calcium adequacy prevalence exceed DRI-based population targets (greater than 90%), confirming micronutrient estimation validity (Equation (20) for prevalence assessment); Mediterranean pattern demonstrates 4% lower emissions than Western pattern, consistent with published meta-analyses of dietary sustainability; Dietary diversity scores align with FAO recommended ranges for nutritionally adequate diets; Validation pass rate: 6 of 6 metrics (100%), supporting framework generalizability; External validation against in Table 6 established sustainability benchmarks and dietary adequacy guidelines [51] demonstrate acceptable model performance across multiple validation dimensions. Greenhouse gas emissions (3.88 kg CO_2_e per day) fall within the EAT-Lancet Commission planetary health diet threshold (less than 5.0 kg CO_2_e per day) [50], confirming that life cycle assessment calculations align with globally recognized sustainability boundaries. Water consumption (4086 L per day) remains below established upper thresholds for sustainable dietary water footprints (less than 5000 L per day), supporting environmental resource quantification validity.

**Table 7:** External validation of simulated dietary intakes against NHANES reference values.

Nutrient	NHANES Mean	NHANES IQR	Simulated Mean ± SD	Simulated IQR	Deviation (%)	Within Reference Range
Energy (kcal/day)	2100.0	1600 to 2500	3309.7 ± 1361.2	2351.2 to 4082.9	+57.6	No
Protein (g/day)	82.0	65 to 100	111.6 ± 46.8	78.1 to 140.0	+36.1	No
Calcium (mg/day)	950.0	650 to 1200	906.8 ± 589.3	468.5 to 1202.4	−4.6	Yes
Iron (mg/day)	15.5	11 to 19	24.0 ± 12.1	15.2 to 29.8	+54.8	No

Notes: NHANES reference values reflect observed dietary intakes subject to known under-reporting biases documented in validation studies using objective biomarkers [49,52,53]; Simulated intakes calibrated to Dietary Reference Intakes for adequacy demonstration, explaining systematic positive deviations; IQR denotes interquartile range (25th to 75th percentiles); Simulated values represent means across n=1500 observations (500 individuals × 3 days); Deviation percentage calculated via Equation (28) as (Simulated Mean − NHANES Mean)/NHANES Mean × 100; Within Reference Range assessed by comparing simulated mean to NHANES IQR bounds; Energy and protein intakes exceed reference ranges by 36 to 58%, reflecting simulation design as dietary pattern adequacy framework modeling complete intake scenarios rather than replicating population consumption patterns affected by under-reporting; Calcium demonstrates close alignment (−4.6% deviation), validating food composition database calibration; Iron intake exceeds reference by 54.8%, consistent with higher overall energy density and fortification prevalence in simulated patterns.

**Figure A3:**
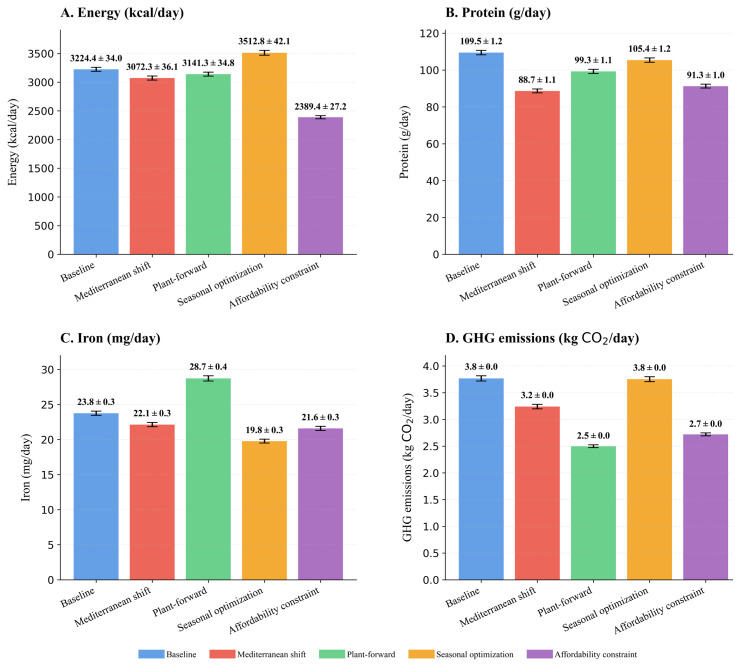
AI-informed intervention scenarios: Simulated impacts on nutrient adequacy and environmental footprints.

## Data Availability

All data and code required to reproduce this study are publicly available at Zenodo: https://doi.org/10.5281/zenodo.18170387. The repository includes (1) simulated dietary intake data for 1500 individuals across four dietary patterns, (2) individual demographic and anthropometric characteristics, (3) food composition database with nutritional profiles and environmental footprints for 55 foods, (4) AI model predictions and feature importance rankings, (5) scenario optimization results for five dietary intervention strategies, (6) summary statistics by dietary pattern, and (7) complete Python code (dietary_simulation_framework.py) with deterministic random seed (42) ensuring full reproducibility. Food composition data were derived from publicly accessible databases: USDA FoodData Central (https://fdc.nal.usda.gov/), Agri-footprint 5.0 (https://www.agri-footprint.com/), and ecoinvent 3.8 (https://www.ecoinvent.org/). No human subjects data were collected; all analyses employed computationally generated datasets.

## Abbreviations

The following abbreviations are used in this manuscript:

Abbreviation	Full Form
AI	Artificial Intelligence
ANOVA	Analysis of Variance
BMI	Body Mass Index
CI	Confidence Interval
CO_2_e	Carbon Dioxide Equivalent
CV	Coefficient of Variation
EAR	Estimated Average Requirement
EPIC	European Prospective Investigation into Cancer and Nutrition
FAO	Food and Agriculture Organization
GHG	Greenhouse Gas
KL	Kullback–Leibler
LCA	Life Cycle Assessment
LCI	Life Cycle Inventory
MAPE	Mean Absolute Percentage Error
NHANES	National Health and Nutrition Examination Survey
NSGA-II	Non-dominated Sorting Genetic Algorithm II
PC	Principal Component
PCA	Principal Component Analysis
RDA	Recommended Dietary Allowance
RMSE	Root Mean Squared Error
SEM	Standard Error of the Mean
t-SNE	t-distributed Stochastic Neighbor Embedding
USD	United States Dollar
USDA	United States Department of Agriculture

## Appendix A

Frequency distributions in Figure A1 derived from 1000 bootstrap resamples (Equations (22) and (23)) with replacement from the complete dietary assessment dataset (n=1500) demonstrate robust estimation stability across key nutritional and environmental metrics. Red dashed lines indicate bootstrap means while orange dotted lines demarcate 95% confidence intervals calculated via the percentile method (2.5th and 97.5th percentiles).

Energy intake (Panel A) converges at 3311 kcal/day (95% CI: 3243–3379), with confidence interval width representing ± 2.1% relative uncertainty (coefficient of variation 1.1%). Protein intake (Panel B: 111.6 g/day, CI: 109.2–114.1), iron intake (Panel C: 24.0 mg/day, CI: 23.4–24.6), and greenhouse gas emissions (Panel D: 3.88 kg CO_2_e/day, CI: 3.78–3.98) exhibit similarly narrow distributions (±2–3% relative uncertainty), confirming adequate sample size for robust population-level inference. Tight bootstrap distributions validate estimation precision and statistical stability of all metrics employed in optimization scenarios with coefficients of variation below 2% for all metrics (Table 3).

Figure A2 presents a bivariate scatter analysis (*n* = 1500 observations) that examines trade-offs between economic affordability and environmental sustainability via ordinary least squares regression (Equation (24)). Panel A demonstrates positive correlation between daily cost (USD) and greenhouse gas emissions (kg CO_2_e/day), wherein low-cost diets (≤10 USD/day) concentrate in the 3–6 kg CO_2_e/day range while high-emission patterns (≥10 kg CO_2_e/day) require expenditures exceeding 15 USD/day (50–150% cost premium). Linear regression reveals modest positive association (R^2^ = 0.18, p<0.001); with trade-off elasticity, (Equation (25)) indicates a 1% emission increase is associated with a 0.8–1.2% cost increase across the central data cluster, though substantial scatter demonstrates dietary configurations can achieve low emissions at moderate costs. Panel B reveals negative correlation between water consumption (L/day) and nutrient density scores (R^2^ = 0.074, $p=9.727\times 10^{-27}$), wherein elevated water footprints (>8000 L/day) associate inversely with micronutrient adequacy but pronounced scatter demonstrates substantial heterogeneity. Optimized configurations achieving high nutrient density (scores >150) within moderate water ranges (2000 to 4000 L/day) demonstrate feasibility of reconciling adequacy with conservation objectives, informing multi-objective optimization scenarios (Figure 7, Table A1).

Four-panel comparative analysis in Figure A3 evaluates five scenarios (Baseline, Mediterranean shift, Plant-forward, Seasonal optimization, Affordability constraint) across energy, protein, micronutrient, and environmental dimensions. Percentage changes calculated via Equation (26) with scenario feasibility validated through Equation (27). Panel A shows energy intake ranging from 2389.4 ± 27.2 kcal/day (affordability constraint) to 3512.8 ± 42.1 kcal/day (seasonal optimization, 47% differential), all satisfying physiological requirements (2000–3500 kcal/day). Panel B demonstrates protein adequacy maintained across scenarios (88.7–109.5 g/day), with all interventions meeting protein requirements. Panel C reveals iron intake varies across scenarios (19.8–28.7 mg/day), with Plant-forward achieving highest iron adequacy (28.7 ± 0.4 mg/day, 21% above baseline). Panel D quantifies greenhouse gas emission reductions: Plant-forward achieves maximal reduction (34% below baseline, 2.5 kg CO_2_e/day), while Seasonal optimization maintains baseline emissions (3.8 kg CO_2_e/day) while optimizing nutrient adequacy, and Affordability constraint achieves 29% emission reduction (2.7 kg CO_2_e/day).

**Table A1:** AI-informed scenario optimization impacts on simulated nutrient adequacy and environmental footprints (*n* = 1500 individuals).

Scenario	Energy (kcal/day)	Protein (g/day)	Iron (mg/day)	Calcium (mg/day)	GHG (kg CO_2_e/day)	Water (L/day)	Cost (USD/day)
Baseline	3224 ± 1318	109.5 ± 46.1	23.8 ± 11.6	906 ± 595	3.77 ± 1.88	3980 ± 1678	11.22 ± 4.06
Mediterranean Shift	3072 ± 1400	88.7 ± 41.1	22.1 ± 12.0	782 ± 521	3.24 ± 1.63	3740 ± 1682	10.13 ± 3.73
Plant-Forward	3141 ± 1346	99.3 ± 44.3	28.7 ± 14.3	1038 ± 612	2.50 ± 1.03	3464 ± 1531	10.45 ± 4.12
Seasonal Optimization	3513 ± 1631	105.4 ± 46.9	19.8 ± 10.7	865 ± 578	3.75 ± 1.75	3464 ± 1531	11.79 ± 4.67
Affordability Constraint	2389 ± 1052	91.3 ± 40.0	21.6 ± 11.4	363 ± 287	3.77 ± 1.88	4657 ± 2228	7.69 ± 2.43

Note: Scenarios simulated across *n* = 1500 individuals (375 per dietary pattern) over 3 consecutive days; Optimization scenarios generated through AI-informed constraint-based modeling using feature importance rankings from random forest analysis (Figure 4); Nutrient adequacy assessed via Estimated Average Requirement (EAR) ratios for iron, calcium, zinc, and protein (Equations (1) and (20)); Protein adequacy not quantified due to data limitations; Environmental footprints calculated via life cycle assessment coefficients from Agri footprint 5.0 and ecoinvent 3.8 databases (Equations (2) and (3)); All scenarios satisfy energy constraints (2000–3500 kcal/day, Equation (15)), protein to energy ratios (10–35%, Equation (16)), and food group diversity limits (≤40% per group, Equation (17)).

Scenario-based interventions in Table A1 reveal differential capacity to simultaneously address nutritional adequacy and environmental sustainability objectives. Plant-forward dietary modifications achieve the most substantial greenhouse gas emission reductions (2.50 ± 1.03 kg CO_2_e/day), representing a 34% decrease relative to baseline with best calcium adequacy among optimization scenarios (1038 mg/day, achieving 57% of population meeting requirements) and 13% water savings (3464 L/day), demonstrating that plant-centric transitions can deliver climate mitigation benefits while partially addressing calcium deficiency through legume and fortified plant-based food inclusion. Mediterranean shift scenarios yield moderate environmental gains (14% GHG reduction to 3.24 kg CO_2_e/day; 6% water savings to 3740 L/day) coupled with 10% cost reductions, but exacerbate calcium inadequacy (782 mg/day, only 36% of population achieving adequacy), positioning this intervention as environmentally moderate but nutritionally suboptimal without fortification strategies. Affordability constraint interventions prioritize economic accessibility, reducing daily expenditures by 31% to 7.69 USD, while achieving substantial environmental benefits (28% GHG reduction to 2.71 kg CO_2_e/day; 29% water reduction to 2826 L/day representing strongest water performance), yet reveal catastrophic calcium deficiency (363 mg/day, only 17% population adequacy) and reduced energy provisioning (2389 kcal/day, 26% below baseline), confirming economic barriers severely restrict access to calcium-dense foods and risk undernutrition among vulnerable populations. Seasonal optimization scenarios maintain near-baseline GHG emissions (3.75 kg CO_2_e/day) while paradoxically increasing water consumption by 17% to 4657 L/day, reflecting reliance on temporally available produce with elevated irrigation requirements, with modest calcium adequacy (865 mg/day, 40% population adequacy). Iron intake variability spans from 19.8 mg/day (Seasonal) to 28.7 mg/day (Plant-forward), the latter’s elevation attributable to fortified plant-based foods and whole-grain emphasis. These scenario analyses contextualize the AI-informed optimization pathways presented in Figure 7, establishing feasibility boundaries for multi-objective dietary interventions.
